# Combinatorial transcription factor profiles predict mature and functional human islet **α** and **β** cells

**DOI:** 10.1172/jci.insight.151621

**Published:** 2021-09-22

**Authors:** Shristi Shrestha, Diane C. Saunders, John T. Walker, Joan Camunas-Soler, Xiao-Qing Dai, Rachana Haliyur, Radhika Aramandla, Greg Poffenberger, Nripesh Prasad, Rita Bottino, Roland Stein, Jean-Philippe Cartailler, Stephen C.J. Parker, Patrick E. MacDonald, Shawn E. Levy, Alvin C. Powers, Marcela Brissova

**Affiliations:** 1 Division of Diabetes, Endocrinology and Metabolism, Department of Medicine, Vanderbilt University Medical Center, Nashville, Tennessee, USA.; 2 Creative Data Solutions, Vanderbilt Center for Stem Cell Biology, Nashville, Tennessee, USA.; 3 Department of Molecular Physiology and Biophysics, Vanderbilt University School of Medicine, Nashville, Tennessee, USA.; 4 Department of Bioengineering, Stanford University, Stanford, California, USA.; 5 Alberta Diabetes Institute and Department of Pharmacology, University of Alberta, Edmonton, Alberta, Canada.; 6 HudsonAlpha Institute for Biotechnology, Huntsville, Alabama, USA.; 7 Imagine Pharma, Devon, Pennsylvania, USA.; 8 Institute of Cellular Therapeutics, Allegheny-Singer Research Institute, Allegheny Health Network, Pittsburgh, Pennsylvania, USA.; 9 Department of Computational Medicine and Bioinformatics, University of Michigan, Ann Arbor, Michigan, USA.; 10 VA Tennessee Valley Healthcare System, Nashville, Tennessee, USA.

**Keywords:** Cell Biology, Endocrinology, Beta cells, Bioinformatics, Diabetes

## Abstract

Islet-enriched transcription factors (TFs) exert broad control over cellular processes in pancreatic α and β cells, and changes in their expression are associated with developmental state and diabetes. However, the implications of heterogeneity in TF expression across islet cell populations are not well understood. To define this TF heterogeneity and its consequences for cellular function, we profiled more than 40,000 cells from normal human islets by single-cell RNA-Seq and stratified α and β cells based on combinatorial TF expression. Subpopulations of islet cells coexpressing *ARX/MAFB* (α cells) and *MAFA*/*MAFB* (β cells) exhibited greater expression of key genes related to glucose sensing and hormone secretion relative to subpopulations expressing only one or neither TF. Moreover, all subpopulations were identified in native pancreatic tissue from multiple donors. By Patch-Seq, *MAFA*/*MAFB*-coexpressing β cells showed enhanced electrophysiological activity. Thus, these results indicate that combinatorial TF expression in islet α and β cells predicts highly functional, mature subpopulations.

## Introduction

Pancreatic islets are cell clusters dispersed throughout the pancreas, composed primarily of endocrine cells that coordinate glucose homeostasis. Islet β cells secrete insulin, which acts to lower blood glucose, and α cells secrete glucagon, which acts to raise blood glucose. In addition to α and β cells, cooperative interaction of less prevalent endocrine cells (δ, γ, and ε) and nonendocrine cell populations in the islet microenvironment, including endothelial cells, macrophages, pericytes (stellate cells), nerve fibers, and immune cells, provide additional signals to modulate islet function (1). Islet α and β cells are characterized by the precise expression of transcriptional and signaling machinery that allows sensing and integration of glucose, nutrient, and neurohormonal signals and proportional response with regulated hormone secretion. Importantly, pancreatic islet dysfunction through impaired insulin and/or glucagon secretion is a hallmark of most forms of diabetes (2–5). Thus, identifying key factors and molecular pathways governing α and β cell identity and function is crucial to understanding, treating, and preventing diabetes.

One set of important molecules governing α and β cell identity and function are islet-enriched transcription factors (TFs) that have been shown to have important roles in islet development and in the maintenance of the islet cell phenotype, particularly in mouse and islet-like cells derived from human stem cells (6–9). Importantly, several islet-enriched TFs have species differences between human and mouse, highlighting the need to closely investigate TFs in human systems (10, 11). For example, members of the Maf TF family show differences in cell-type distribution and timing of expression (12, 13). Such TFs interact in complexes and networks to exert broad control over cellular processes, making them foundational regulators of cell states. In fact, in addition to their coordinated role in islet cell development, loss or misexpression of key TFs has been highlighted in numerous forms of diabetes (14–17).

Importantly, with advances in scientific methodologies, it has been increasingly recognized that islet cells are heterogeneous. This is particularly apparent in β cells: recent work has highlighted human β cell heterogeneity in function (18), cell-surface protein expression (19, 20), and transcriptomic profile (21, 22). In contrast, heterogeneity within human α cells has been much less studied. Given the central role for islet-enriched TFs in regulating cell states, potential heterogeneity in these TFs may represent distinct cellular states with broad implications for human islet biology and diabetes.

RNA-Seq has been an essential technology to broadly characterize islet gene expression in an unbiased manner. Hallmark gene transcripts and gene pathways have been analyzed at the whole-islet level (23, 24) and in a cell type–specific manner using FACS with either cell-surface markers on live cells or intracellular proteins in fixed and permeabilized cells to obtain purified α and β subpopulations (25–27). However, these approaches provide limited ability to assess heterogeneity within a given cell type. To address this, single-cell RNA-Seq (scRNA-Seq) is an exciting and evolving technology that can be used to understand cell-type heterogeneity and has begun to be applied to human islets (18, 28–32). While the magnitude of high-resolution data from these studies is exciting, there are also important technical challenges inherent to the small scale of input material (33, 34), highlighting the importance of a robust comparison between bulk and scRNA-Seq. Further, it remains unclear how α and β cells identified by protein-based methods (e.g., FACS) compare with cells characterized by the clustering approach applied in scRNA-Seq that arranges cells by transcriptional similarity.

To investigate how heterogeneity of islet-enriched TFs in human islets relates to islet function, we focused on 3 TFs, namely *ARX*, *MAFB*, and *MAFA*. ARX and MAFB are enriched in islet α cells, as are MAFA and MAFB in β cells, and all 3 play important roles in islet cell development and disease as suggested by existing bulk RNA-Seq data sets (10, 17, 26, 27). Since our goal was to understand single-cell heterogeneity, we translated findings from a bulk context to a single-cell context by systematically analyzing the same islet preparation by both approaches to establish congruency between bulk and scRNA-Seq methods. Finally, we generated an scRNA-Seq data set of over 40,000 islet cells from adult donors, which included endocrine, immune, and endothelial cell populations, that is accessible through a user-friendly web portal. This data set provided sufficient cell numbers to classify α and β cells into subgroups based on combinatorial *ARX*/*MAFB* and *MAFA*/*MAFB* expression, respectively, and allowed us to identify key correlates to α and β cell function. We further validated the existence of these cell populations within human pancreatic tissue in situ and linked *MAFA*/*MAFB* transcriptional heterogeneity of human β cells to their electrophysiological properties.

## Results

**Figure 1:**
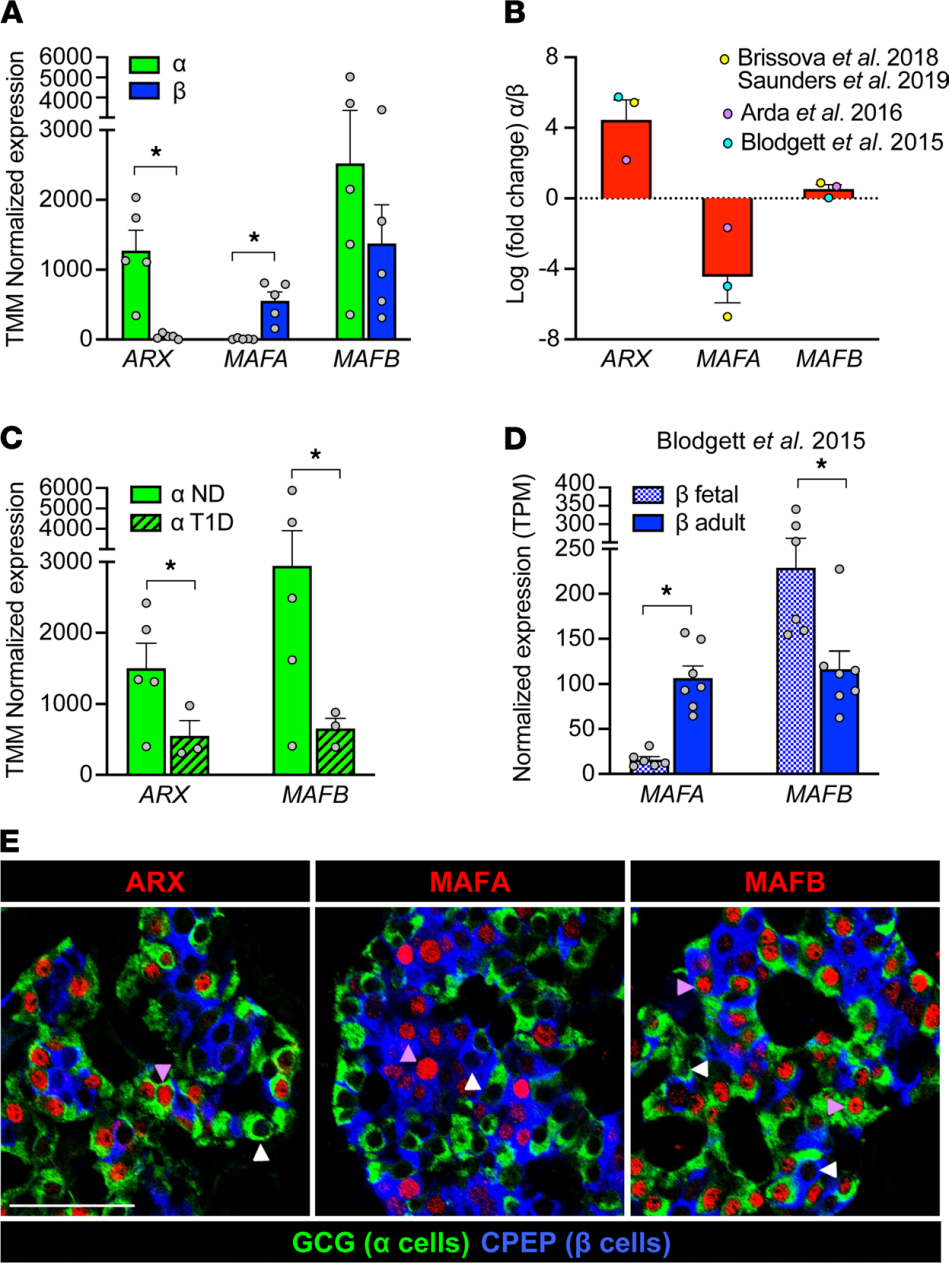
Bulk RNA-Seq and immunohistochemistry data highlight unique expression patterns of transcription factors ARX, MAFA, and MAFB in human α and β cells. (**A**–**D**) Normalized expression values (**A**, **C**, and **D**) and fold change (**B**) of *ARX*, *MAFA*, and *MAFB* in previously published bulk RNA-Seq data sets from α cells (green) and β cells (blue). Data in **A** is from Brissova et al. (17) and Saunders et al. (27) (*n =* 5 donors); additional data sets from Arda et al. (10) (*n =* 5 donors) and Blodgett et al. (26) (*n =* 7 donors) are included in **B**. See also Supplemental Figure 1, A and B. (**C**) Expression of *ARX* and *MAFB* is decreased (*ARX* fold change: –2.7; *MAFB*: –3.4) in α cells from donors with type 1 diabetes (T1D) compared with nondiabetic (ND) donors (17). (**D**) Expression of *MAFA* is increased (fold change: 7.1) in adult β cells compared with fetal β cells, while *MAFB* is decreased (fold change: –2.0) (26). All data shown as mean + SEM; symbols represent individual donors (**A**, **C**, and **D**) or average value per data set (**B**). Asterisks indicate significant (adjusted *P* value < 0.05) fold change of α versus β in **A** and **B**, T1D versus ND in **C**, and adult versus fetal in **D**. (**E**) Immunohistochemical staining of pancreatic sections from a nondiabetic adult (55 years, Supplemental Table 4), showing specificity of ARX, MAFA, and MAFB (red) in α cells (GCG; green) and β cells (CPEP; blue). Arrowheads indicate cells negative (white) or positive (purple) for transcription factors; scale bar: 50 μm. See also Supplemental Figure 1, C and D.

*Transcriptional and immunohistochemical profiling of humanαandβcells suggests a role for key TFs — ARX, MAFA, and MAFB — in islet cell development and disease*. In vivo and in vitro studies have helped identify TFs with cell-specific expression patterns in islets. In α cells, aristaless related homeobox (ARX) factor is essential for α cell differentiation and function, a finding that has been confirmed in human α cells (8, 35–37). Indeed, *ARX* transcripts are heavily enriched in α cells (Figure 1, A and B, refs. 12, 38, 39, and Supplemental Figure 1, A and B; supplemental material available online with this article; https://doi.org/10.1172/jci.insight.151621DS1). Of note, α cells from donors with type 1 diabetes (T1D) showed decreased *ARX* expression compared with α cells from nondiabetic donors (Figure 1C), indicating that this factor may contribute to impaired glucagon secretion observed in T1D (17, 18, 40).

MAFA is a bona fide β cell factor exerting direct control over insulin expression as well as key components of glucose-stimulated insulin secretion, and it is expressed relatively late in β cell development, making it a commonly used marker of fully mature β cells (41–43). MAFA is thought to play a broadly similar role in adult mouse and human β cells, and existing RNA-Seq data sets underscore its β cell specificity (Figure 1, A and B and Supplemental Figure 1, A and B). MAFA is clearly present in adult β cells, but its expression actually does not peak until several years after birth, as illustrated by previous histological studies (12) and transcriptomic profiles of β cells from fetal versus adult donors (Figure 1D and ref. 26). These data temporally correlate increased MAFA levels with the acquisition of increased glucose sensitivity (44–46), suggesting that MAFA plays a role in β cell maturation and function.

In contrast to ARX and MAFA, MAFB is expressed by both α and β cells (Figure 1, A and B and Supplemental Figure 1, A and B) and shows significant species differences: it is retained in human β cells during adulthood, whereas in rodents it becomes restricted to α cells in the early postnatal period (11). Of note, the MAF factors are thought to be capable of forming both homo- and heterodimers (47), providing an opportunity for synergy between MAFA and MAFB in β cells. In α cells, MAFB is known to directly bind to the *GCG* promoter to regulate glucagon expression (32), rendering it an important regulator of α cell function. Like *ARX*, *MAFB* is reduced in α cells from donors with T1D (Figure 1C).

The unique and dynamic expression patterns of *ARX*, *MAFA*, and *MAFB* demonstrated by bulk RNA-Seq (Figure 1, A–D, and Supplemental Figure 1, A and B) suggest that these TFs are linked to key aspects of α and β cell function. However, our analysis of their special distribution in adult human pancreatic tissue revealed that not all α or β cells in a given islet express them (Figure 1E and Supplemental Figure 1, C and D). Thus, to further understand the role of these TFs, we sought to determine the cell-to-cell variability that cannot be discerned from a pooled cell population profiled by bulk RNA-Seq. Given the known importance of TFs in regulating cellular processes, we hypothesized that TF heterogeneity at the single-cell level could define α or β cell subtypes with different functional properties.

**Figure 2:**
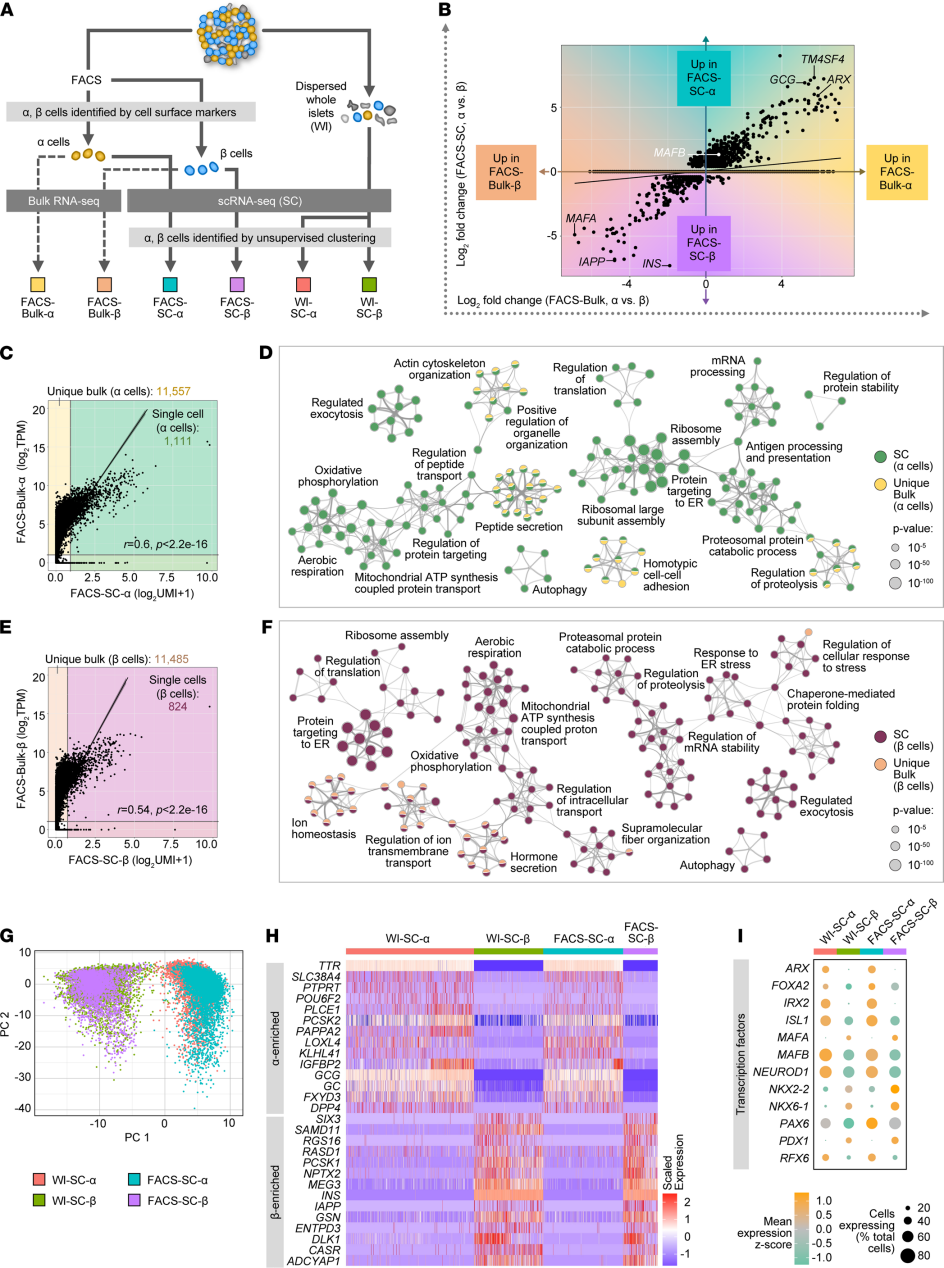
Gene expression profiles of α and β cells defined by scRNA-Seq are largely concordant with those obtained by bulk RNA-Seq. (**A**) Schematic depicting comparison of sorted human α and β cells profiled by bulk (FACS-Bulk) and single-cell (FACS-SC) RNA-Seq (*n =* 1, 39-year-old donor), as well as single α and β cells identified by cell surface markers (FACS-SC) compared with those from dispersed whole islets (WI-SC) identified by unsupervised clustering (*n =* 2, 14- and 39-year-old donors). (**B**) Genes differentially expressed (log_2_ fold change) between α and β cells as assayed by scRNA-Seq (*y* axis) and bulk RNA-Seq (*x* axis). (**C**–**F**) Gene expression and associated gene ontology term enrichment for α (**C** and **D**) and β (**E** and **F**) cells by bulk and scRNA-Seq. Scatterplots (**C** and **E**) show average expression (unique molecular identifier [UMI] counts) from scRNA-Seq (7269 α; 2511 β) compared with TPM normalized expression of bulk RNA-Seq (10,000 cells/sample) from corresponding populations. Only genes log_2_TPM > 1 (bulk) and UMI > 1 (single cell) were considered to assess gene detection; *r* is Pearson’s coefficient and *P* is significance from *t* test statistic. Metascape (49) network visualizations (**D** and **F**) show enriched ontology terms from genes detected by scRNA-Seq (“SC”) and 1000 genes uniquely detected by bulk RNA-Seq (“Unique Bulk”) that were most differentially expressed in each cell type. Colors correspond to shaded regions of **C** and **E**. (**G**) Principal component analysis (PCA) of sorted α and β cells identified by cell-surface marker expression (FACS-SC) and those derived from dispersed whole islets and identified by unsupervised clustering (WI-SC). (**H**) Heatmap showing variable expression of known α cell– and β cell–enriched markers within and between each sample. (**I**) Relative expression of transcription factors across samples; dot size indicates the percentage of cells with detectable transcripts and color indicates gene’s mean expression by *z* score.

*Gene expression profiles ofαandβcells defined by scRNA-Seq are largely concordant with those obtained by bulk RNA-Seq*. To translate gene expression findings from bulk RNA-Seq into the single-cell context, we systematically analyzed FACS-purified α and β cells (17, 27, 48) from a healthy 39-year-old donor by the 2 approaches in parallel (Figure 2A, Supplemental Figure 2A, and Supplemental Table 1). Approximately 10,000 cells for each cell type were pooled to generate bulk RNA-Seq libraries (“FACS-Bulk-α” and “FACS-Bulk-β”) and 10,000 or more α cells and β cells were processed for scRNA-Seq to capture 6371 and 1190 single cells after quality control (“FACS-SC-α” and “FACS-SC-β”), respectively.

Because bulk RNA-Seq and scRNA-Seq involve different chemistries that may bias direct comparisons of gene expression levels, we initially assessed relative differences by looking at differential expression between α and β cells profiled by each approach (FACS-Bulk-α vs. FACS-Bulk-β compared with pooled data from FACS-SC-α vs. FACS-SC-β). Genes differentially expressed in both data sets were highly correlated (*r* = 0.91, *P* < 2.2 × 10^–16^) and showed the expected enrichment of β cell–specific genes (e.g., *INS, IAPP, MAFA*) as well as α cell–specific genes (*GCG,* *TM4SF4, ARX*) (Figure 2B). Importantly, there were very few differentially expressed genes that were regulated in opposite directions (Figure 2B), suggesting that trends in gene expression were consistent between the 2 methods. In addition, gene expression in each cell type was quite concordant between bulk and scRNA-Seq (*r* > 0.5, *P* < 2.2 × 10^–16^); however, as expected, overall gene coverage was much greater in bulk RNA-Seq (Figure 2, C and E). To determine what biological information this extra coverage provided, we visualized enriched pathways by gene ontology (GO) and Kyoto Encyclopedia of Genes and Genomes (KEGG) for genes detected by scRNA-Seq (“SC”) as well as for genes uniquely captured by bulk RNA-Seq (“Unique Bulk”) that were most specific to each cell type (Figure 2, D and F, Supplemental Figure 2, F and G; and ref. 49). Enriched processes were highly related and integrated, highlighting important islet cell functions (GO: ion homeostasis, regulated exocytosis, autophagy, etc.; KEGG: glycolysis, insulin secretion, cAMP signaling, etc.). Taken together, these data indicate that although bulk RNA-Seq captured a greater breadth of genes, scRNA-Seq analysis provided data for a similarly broad and comprehensive set of pathways and processes specific to α and β cell biology.

We next asked whether gene expression profiles of FACS-purified α and β cells (17, 27, 48) were similar to those identified by unsupervised clustering (identification of cells after sequencing). Islet cells from 2 healthy donors were profiled by scRNA-Seq either directly after dispersion from whole islets (WIs; “WI-SC-α” and “WI-SC-β”) or after FACS purification (“FACS-SC-α” and “FACS-SC-β;” Figure 2A and Supplemental Table 1) with a similar gene capture across all 4 cell populations (Supplemental Figure 2B). Principal component analysis (PCA) indicated that overall variance was not governed by cell identification approach but rather cell-type differences (Figure 2G) driven by known α and β cell genes (e.g., *GCG*, *SLC7A2*, *INS*, *PCSK1*) as well as markers not extensively studied in islets (e.g., *RGS4*, *FXYD3*, *MEG3*, *HADH*; Supplemental Figure 2C). In addition, gene expression profiles of FACS-α and FACS-β samples showed strong linear correlation (*r* = 0.99, *P* < 2.2 × 10^–16^) with WI-α and WI-β samples, respectively (Supplemental Figure 2, D and E). Visualization of canonical α and β cell markers (Figure 2H) highlighted that cell-cell heterogeneity was apparent regardless of cell identification method. Finally, key islet-enriched TFs showed consistency between WI and FACS samples for each cell type, both in magnitude of expression (*z* score, indicated by color) and in number of cells expressing the factor (dot size) (Figure 2I). These results indicate that a) cell sorting did not appreciably alter the transcriptional profile of α and β cells and b) post hoc identification of cell types by unsupervised clustering was consistent with identification by cell-surface proteins. Thus, both approaches are suitable for investigating TF heterogeneity.

**Figure 3:**
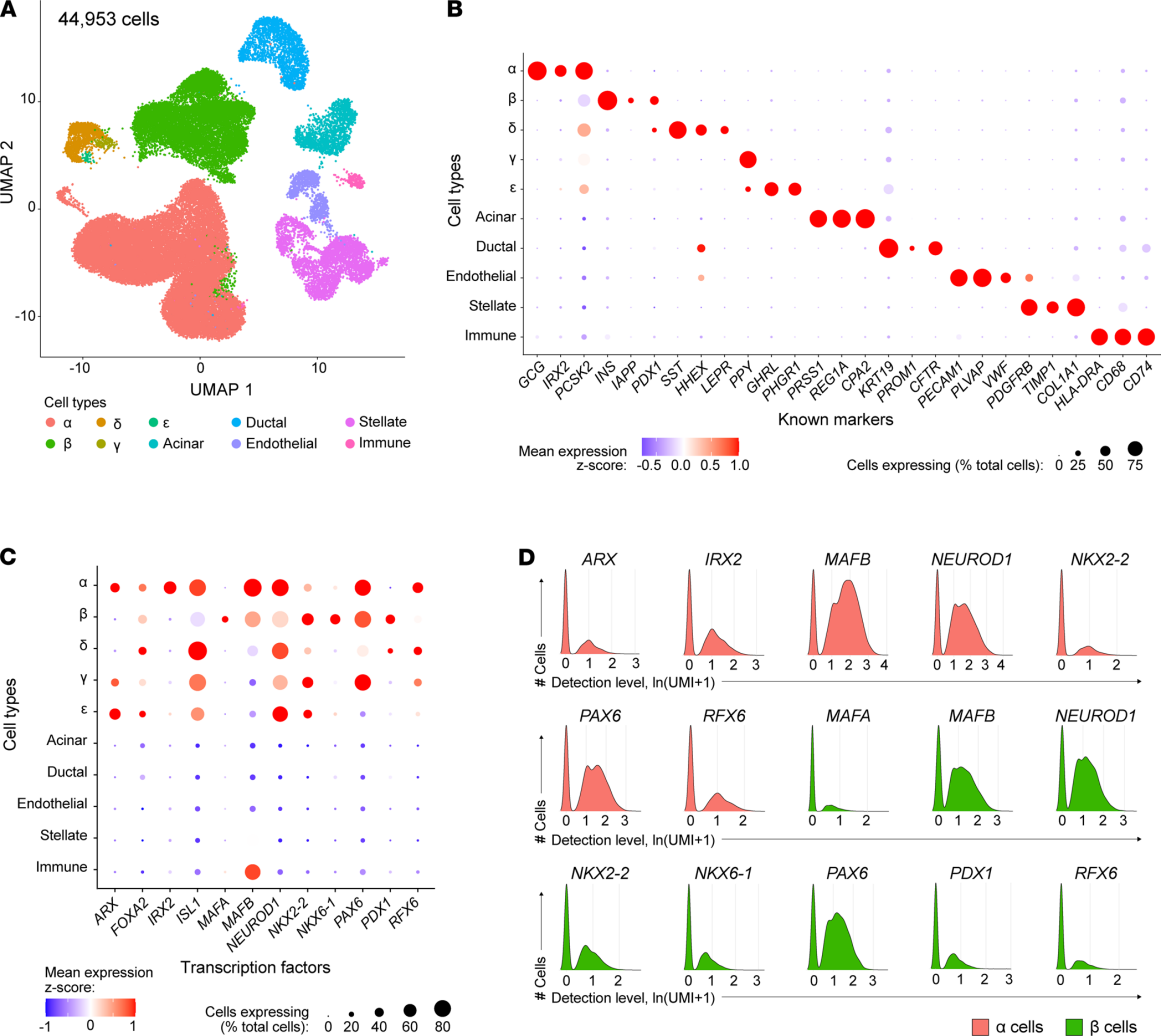
Transcription factor expression in human pancreatic islets by scRNA-Seq. (**A**) UMAP visualization of 44,953 pancreatic islet cells from *n =* 5 islet preparations, identified by unsupervised clustering; cell populations include β (24%), α (54%), δ (2.5%), ε (0.08%), acinar (3.3%), ductal (4.7%), endothelial (2.2%), stellate (7.7%), and immune cells (0.5%). Cell clusters were annotated using known gene markers (Supplemental Table 2). Populations of γ and ε cells could not be resolved from the δ cell cluster; thus, these populations were manually selected using the “CellSelector” function to identify cells positive for *PPY* and *GHRL*, respectively. Libraries were sequenced at approximately 80,000 reads/cell yielding a median of 2365 genes per cell. (**B**) Dot plot showing relative expression of cell-type markers to validate cell-type annotation after unsupervised clustering. (**C**) Dot plot showing relative expression of transcription factors across all cell types. In **B** and **C**, dot size indicates the percentage of cells with detectable transcripts; color indicates gene’s mean expression *z* score. (**D**) Detected levels of common transcription factors expressed in α and β cells expressed as natural log (unique molecular identifiers per 10,000 + 1).

*scRNA-Seq reveals heterogenous TF expression inαandβcells*. One major advantage of scRNA-Seq is its ability to dissect heterogeneous cell composition within and across cell types. However, because some subpopulations are relatively rare, robust data sets are required to sufficiently characterize these populations. In this study, we obtained 44,953 high-quality single-cell transcriptomes of handpicked islets from *n =* 5 healthy donors with robust dynamic insulin and glucagon secretion profiles characterized by perifusion to ensure healthy and functional cells were being assessed (Supplemental Table 1 and Supplemental Figure 3A). Graph-based unsupervised clustering (50) reliably detected major endocrine cell types (α, β, δ) and acinar, ductal, stellate, endothelial, and immune cells (Figure 3A). Clusters were annotated to identify cell types, including rare populations such as γ and ε, using markers listed in Supplemental Table 2, and identified cell types were represented in each donor (Supplemental Figure 3B). Cell populations were confirmed by the specific expression of additional known identity markers (Figure 3B). Within cell types, the expected clustering by individual donor (Supplemental Figure 3C) was apparent. To facilitate the exploration of this robust single-cell data set, we created a web application that allows one to browse single-cell gene expression by both cell type and donor (Supplemental Figure 3D).

To investigate the cell-specific signatures of human α and β cells, we analyzed expression patterns of canonical islet-enriched TFs. *PAX6*, *RFX6*, *NEUROD1*, and *NKX2-2* were detected in all endocrine cell types, whereas *PDX1*, *NKX6-1*, and *MAFA* were enriched in β cells; *IRX2* was specifically detected in α cells; and *ARX* was detected in α, γ, and ε cells, consistent with previous single-cell studies (refs. 28, 29, 50 and Figure 3C). *PAX6*, *NEUROD1*, and *MAFB* were among the most prevalent endocrine factors, detected in more than 75% of α and β cells (Figure 3C). Of particular interest, *MAFB* — known in humans to be expressed in both α and β cells — was also enriched in the immune cell population, which had been overlooked in previous studies because of low abundance of immune cells in isolated islets. Interestingly, we noticed that each of these key TFs had a bimodal distribution, meaning there was a clear subpopulation of cells without detection of each factor (Figure 3D), consistent with our observations for MAFA, MAFB, and ARX in pancreas tissue (Figure 1E). Ranges in the number of detected genes per cell for cells expressing low (natural log [unique molecular identifiers per 10,000 + 1] < 0.5) and high (natural log [unique molecular identifiers per 10,000 + 1] > 0.5) *MAFA*, *MAFB*, and *ARX* were comparable, with the majority of cells having more than 1000 detected genes (Supplemental Figure 3E). These results indicate that the bimodality of TF distribution is unlikely to be due to cells having a high dropout rate; there was also no evidence that cell-cycle state contributed to the stratification (Supplemental Figure 3F). Given the crucial role that islet-enriched TFs play in islet cell identity and function, particularly when acting in TF regulatory networks, we thus hypothesized that combinations of key TFs would identify important islet cell subtypes.

**Figure 4:**
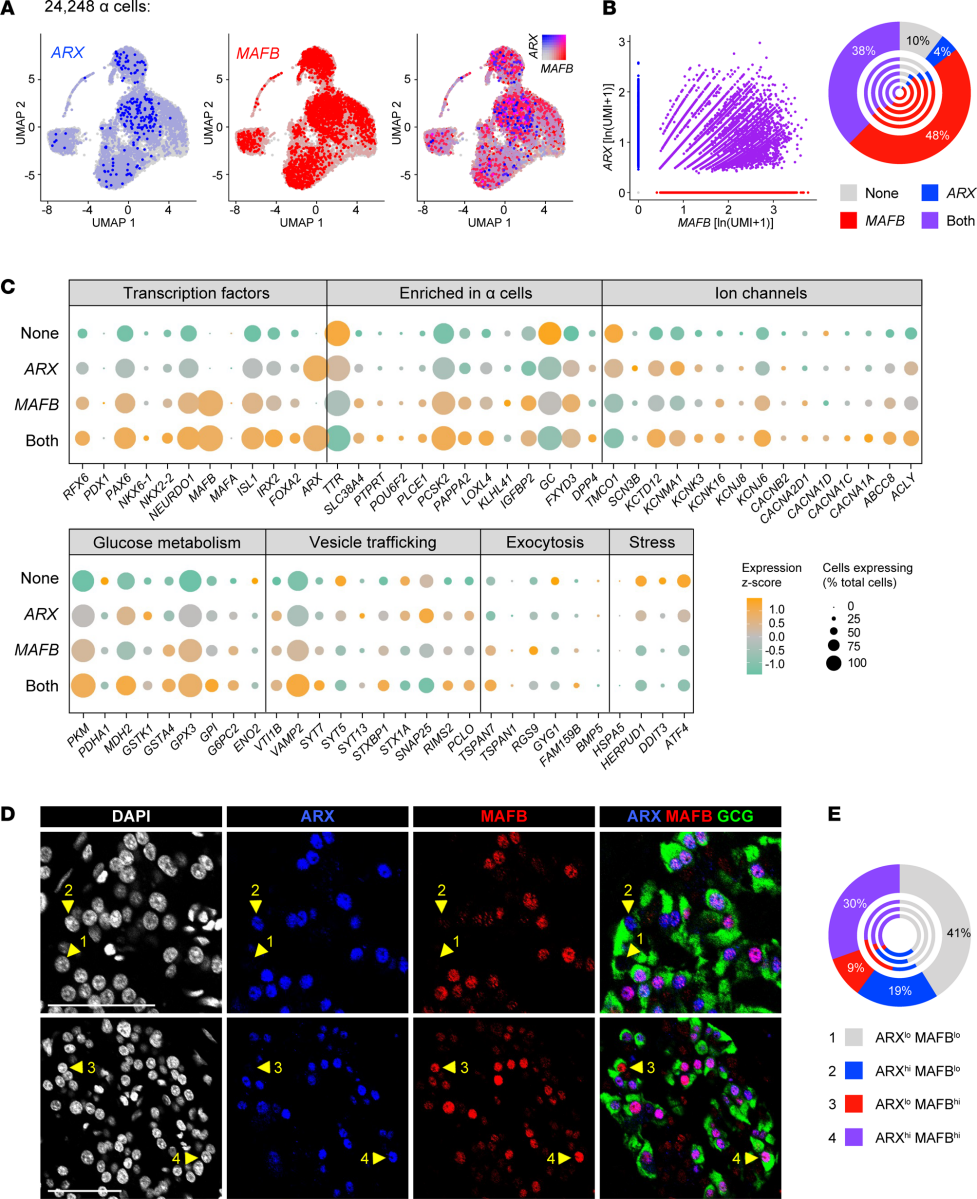
Heterogeneity of *ARX* and *MAFB* expression in α cells by scRNA-Seq correlates with expression of key functional genes. (**A**) UMAP visualization of 24,248 α cells (*n =* 5 donors) pseudocolored to show detected expression, from left to right, of *ARX* (blue); *MAFB* (red); and both *ARX* and *MAFB* with 0.5 color threshold scale. (**B**) Scatterplot on the left is depicting 4 distinct α cell populations based on detected expression (natural log of unique molecular identifiers per 10,000 + 1) of *ARX* and *MAFB*: those expressing neither factor (10%), those expressing only *ARX* (4%) or only *MAFB* (48%), and those coexpressing *ARX* and *MAFB* (38%). Chart on the right shows cell populations by donor, with the outermost circle reflecting totals. (**C**) Dot plot showing the relative expression of selected genes related to α cell identity, ion flux, glucose metabolism, vesicle trafficking, exocytotic machinery, and cellular stress of the 4 α cell populations in **B**. Dot size indicates the percentage of α cells with detectable transcripts; color indicates the gene’s mean expression *z* score. See Supplemental Figure 5 for comparison with other single-cell studies. (**D**) Immunohistochemical staining of ARX (blue) and MAFB (red) in glucagon-expressing (GCG-expressing) α cells (green) of a nondiabetic adult (55 years, Supplemental Table 4). Numbered arrowheads indicate the presence of 4 α populations: 1, ARX^lo^ MAFB^lo^; 2, ARX^hi^ MAFB^lo^; 3, ARX^lo^ MAFB^hi^; 4, ARX^hi^ MAFB^hi^. (**E**) Quantification of α cell populations shown in **D** (*n =* 2369 α cells). Outermost circle represents composite count and inner circles represent α cells from each of *n =* 3 donors (see also Supplemental Figure 5B).

*Heterogeneity of ARX and MAFB expression inαcells by scRNA-Seq predicts expression of keyαcell functional genes*. Since both ARX and MAFB are downregulated in α cells from donors with T1D (17), we tested the hypothesis that these factors cooperatively regulate α cell function. We first confirmed heterogeneous *ARX* and *MAFB* expression in α cells from all 5 donors (Figure 4A). Of 24,248 total α cells, we identified populations of α cells in which neither *ARX* nor *MAFB* was detected (“None”; 10%), populations where only *ARX* or only *MAFB* was detected (4% and 48%, respectively), and a population with codetection of both *ARX* and *MAFB* (“Both”; 38%) that were relatively stable across all 5 donors (Figure 4B). For these 4 populations, we investigated expression of other islet-enriched TFs, α cell–enriched genes, and genes related to ion flux, glucose metabolism, vesicle trafficking, exocytosis, and cell stress (Figure 4C and Supplemental Figure 4). Interestingly, we observed that numerous α cell–enriched TFs (*RFX6*, *PAX6*, *NEUROD1*, *ISL1*, *IRX2*) and genes related to nutrient sensing or glucagon secretion (*ACLY*, *PKM*, *GSTA4*, *GPX3*, *G6PC2*, *KCTD12*, *KCNK16*, *KCNJ6*, *ABCC8*) were elevated in α cells in which *MAFB* and *ARX* were codetected compared with the other populations, whereas genes related to cell stress (*DDIT*, *ATF4*) were highest in the “None” group, suggesting that the presence of both factors may support increased metabolic activity and glucagon secretory capacity. To confirm these findings, we analyzed 3 additional scRNA-Seq data sets of human islets that utilized different single-cell technologies (18, 28, 29) and found the results to be consistent (Supplemental Figure 5A).

We next asked whether *ARX*/*MAFB* heterogeneity existed at the protein level given the known differences that exist between transcript and protein expression (51). To assess this, we performed immunohistochemical analysis of ARX and MAFB on pancreatic tissue sections from nondiabetic donors (Figure 4D and Supplemental Figure 5B). Cells were classified by automated algorithm for “low” or “high” ARX and MAFB immunofluorescence, setting an intensity threshold that remained consistent across all islets from a given tissue. By this measure, all 4 combinations of ARX/MAFB-expressing α cells were detected in each donor evaluated: ARX^lo^ MAFB^lo^ (41%), ARX^hi^ MAFB^lo^ (19%), ARX^lo^ MAFB^hi^ (9%), and ARX^hi^ MAFB^hi^ (30%; Figure 4E). Taken together, our results indicate the presence of α cell subpopulations classified according to unique and conjunctional expression of ARX and MAFB and suggest that combined expression of these 2 markers likely identified highly functional and mature α cells.

**Figure 5:**
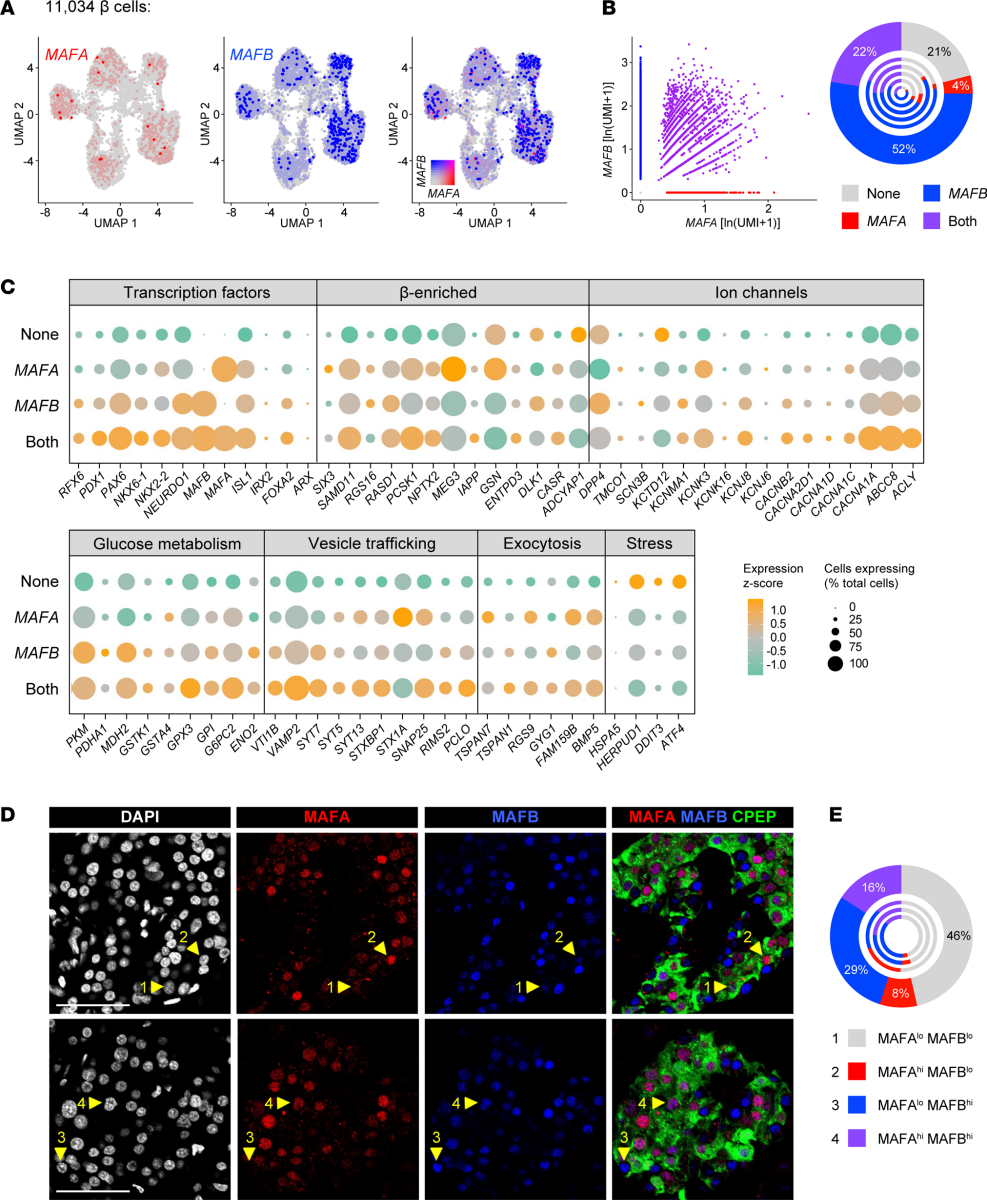
Heterogeneity of MAFA and MAFB expression in β cells by scRNA-Seq correlates with expression of key genes involved in β cell function. (**A**) UMAP visualization of 11,034 β cells (*n =* 5 donors), pseudocolored to show detected expression, from left to right, of *MAFA* (red); *MAFB* (blue); and both *MAFA* and *MAFB* with 0.5 color threshold scale. (**B**) Scatterplot on the left depicts 4 distinct β cell populations based on detected expression (natural log of unique molecular identifiers per 10,000 + 1) of *MAFA* and *MAFB*: those expressing neither factor (22%), those expressing only *MAFA* (4%) or only *MAFB* (52%), and those coexpressing *MAFA* and *MAFB* (22%). Chart on the right shows cell populations by donor, with the outermost circle reflecting totals. (**C**) Dot plot showing the relative expression of selected genes related to β cell identity, ion flux, glucose metabolism, vesicle trafficking, exocytotic machinery, and cellular stress of the 4 β cell populations in **B**. Dot size indicates the percentage of β cells with detectable transcripts; color indicates the gene’s mean expression *z* score. See Supplemental Figure 7 for comparison to other single-cell studies. (**D**) Immunohistochemical staining of MAFA (red) and MAFB (blue) in C-peptide–expressing (CPEP-expressing) β cells (green) of a nondiabetic adult (55 years, Supplemental Table 4). Numbered arrowheads indicate the presence of 4 populations: 1, MAFA^lo^ MAFB^lo^; 2, MAFA^hi^ MAFB^lo^; 3, MAFA^lo^ MAFB^hi^; 4, MAFA^hi^ MAFB^hi^. (**E**) Quantification of β cell populations shown in **D** (*n =* 2566 β cells). Outermost circle represents composite count and inner circles represent β cells from each of *n =* 3 donors (see also Supplemental Figure 7B).

*βCells coexpressing MAFA and MAFB exhibit characteristics of enhanced secretory function*. Given the ability of MAFA and MAFB to heterodimerize (47) and the unique expression changes during β cell maturation (10, 12, 26), we hypothesized that MAFA and MAFB coexpression represents a unique subpopulation of human β cells. To test this, we resolved 11,034 β cells into subgroups in which only *MAFA* or only *MAFB* was detected (4% and 52%, respectively), both *MAFA* and *MAFB* were detected (“Both”; 22%), and neither *MAFA* nor *MAFB* was detected (“None”; 21%; Figure 5, A and B). We assessed these groups for the same set of key cellular identity and functional genes described above for α cells, and we saw a general trend of increased expression of key functional genes with dual *MAFA* and *MAFB* expression (Figure 5C and Supplemental Figure 6). Specifically, numerous genes related to cell identity (*PDX1*, *PAX6*, *NEUROD1*, *ISL1*, *PCSK1*, *IAPP*), glucose metabolism (*ACLY*, *G6PC2*, *GPX3*), ion channels (*ABCC8*, *KCNJ6*), and exocytosis (*VAMP2*, *SYT7*, *PCLO*, *TSPAN7*, *RGS9*, *FAM159B*, *BMP5*) were all increased in MAFA- and MAFB-coexpressing cells compared with other subgroups. In contrast, stress genes (*HSPA5*, *HERPUD1*, *DDIT3*, *ATF4*) were either significantly reduced in the coexpression group or significantly elevated in the “None” group. These expression patterns indicate that presence of both factors may be crucial for increased metabolic activity and insulin secretion. Analysis of 3 independent single-cell studies of human islets utilizing other platforms (18, 28, 29) confirmed these results (Supplemental Figure 7A). The presence of β cell MAFA/MAFB heterogeneity at the protein level (MAFA^lo^ MAFB^lo^, 46%; MAFA^hi^ MAFB^lo^, 8%; MAFA^lo^ MAFB^hi^, 29%; MAFA^hi^ MAFB^hi^, 16%) was validated by immunofluorescence in pancreatic sections, where cells representative of all 4 populations were identified in each of multiple nondiabetic donors (Figure 5, D and E and Supplemental Figure 7B).

**Figure 6:**
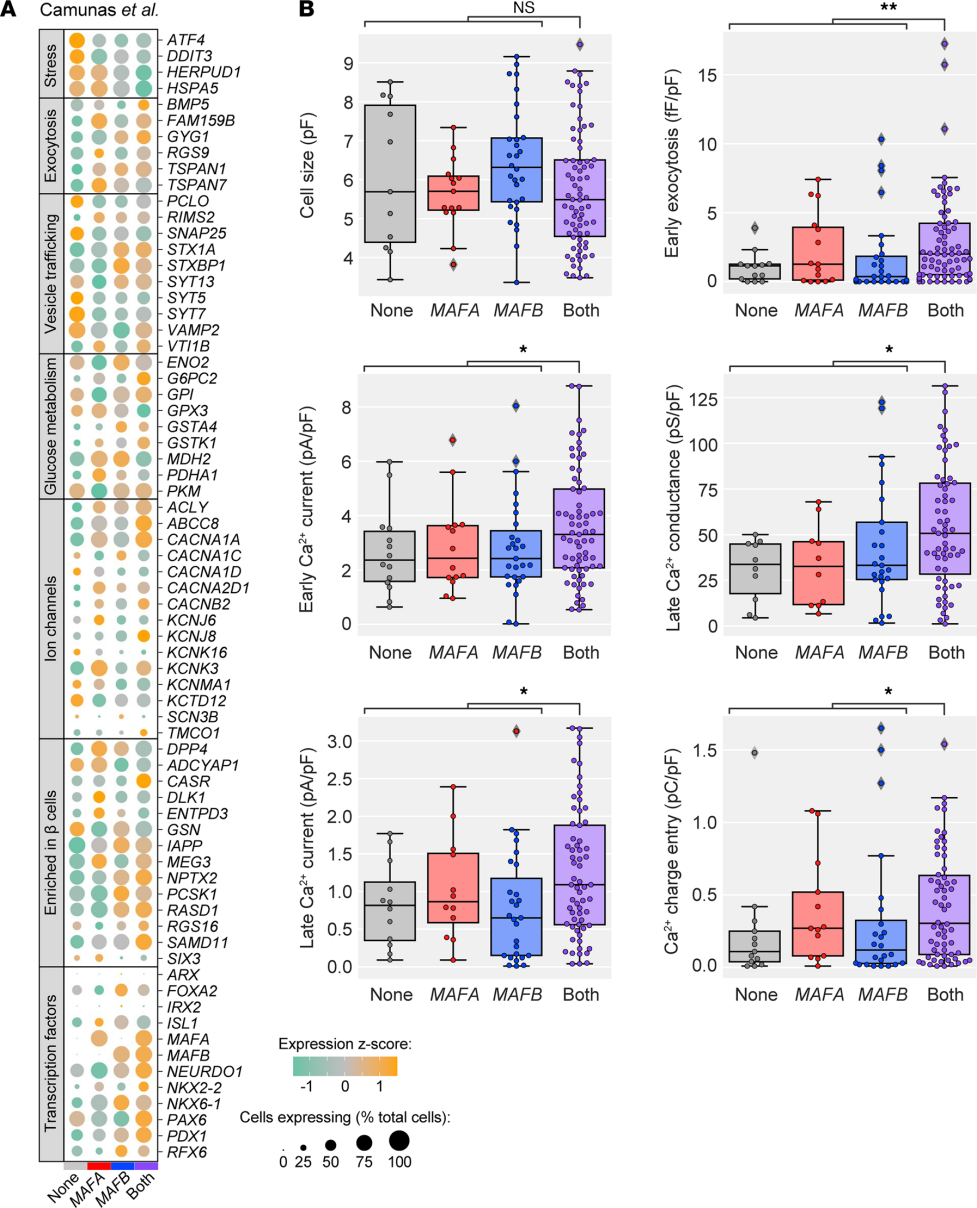
β Cells coexpressing *MAFA* and *MAFB* have enhanced electrophysiological activity compared with β cells expressing one or neither factor. (**A**) Dot plot showing the relative expression of selected genes in β cells expressing neither *MAFA* nor *MAFB*, those expressing only *MAFA* or only *MAFB*, and those coexpressing *MAFA* and *MAFB*, based on data from Camunas et al. (18). Dot size indicates the percentage of cells with detectable transcripts; color indicates gene’s mean expression *z* score. (**B**) Electrophysiological function in *MAFA*- and *MAFB*-expressing β cell subpopulations. Significantly higher Ca^2+^ currents and exocytosis were observed for β cells expressing both *MAFA* and *MAFB* with similar cell size across all subpopulations. Mann-Whitney test adjusted for multiple hypothesis testing with Benjamini-Hochberg (BH) procedure; *P < 0.05; **P < 0.01.

To determine whether the β cell subpopulation in which both *MAFA* and *MAFB* were detected, enriched for numerous genes related to metabolism and hormone secretion, had functionally relevant consequences compared with other β cells, we utilized human Patch-Seq data from Camunas et al. (18). Transcriptomes from 194 β cells within this data set (Figure 6A) showed high similarity with our larger data set of 11,034 β cells (Figure 5C and Supplemental Figure 7A). In addition to producing an mRNA profile, the Patch-Seq approach captured an electrophysiological profile of each cell, generating linked data on cell size, exocytosis, and ion channel currents. In agreement with transcriptome data, β cells with detection of both *MAFA* and *MAFB* showed increased electrophysiological activity across several parameters, including early exocytosis, early and late Ca^2+^ current, and late Ca^2+^ conductance when compared with cells in which *MAFA* only, *MAFB* only, or neither factor was detected (Figure 6B). Of note, *MAFA*/*MAFB* β cells were comparable in size to those expressing only one or neither factor, suggesting that neither the transcriptomic data nor the elevated electrophysiological activity can be attributed to larger cells expressing more genes (Figure 6B). Thus, these data provide strong support that heterogeneous populations of β cells on the basis of combinatorial MAFA/MAFB expression exist and that coexpression of both factors marks β cells with elevated function.

## Discussion

By transcriptional profiling and assessment of protein expression at the single-cell level, we found that several key islet-enriched TFs important for α and β cell maturity and function had a heterogenous expression pattern within normal human islet cells. To unravel the functional consequences of this heterogeneity in TF expression, we systematically analyzed the same islet preparation by bulk and scRNA-Seq approaches and established congruency between the 2 methods. Capitalizing on our large scRNA-Seq data set, we stratified α and β cells based on differential or combined detection of key TFs (*ARX*/*MAFB* in α cells; *MAFA*/*MAFB* in β cells) that are known to act cooperatively. We found that coexpression of these TF combinatorial pairs predicted greater expression of genes related to glucose metabolism, ion flux, and hormone secretion, including known α and β cell functional markers and those not extensively studied in islets. Importantly, we identified subpopulations with TF heterogeneity at the protein level by spatial analysis of normal human tissue and demonstrated, using Patch-Seq, greater electrophysiological activity in β cells coexpressing *MAFA* and *MAFB*. These results suggest that combinatorial expression of key islet TFs defines and predicts highly functional and mature α and β cells.

Bulk RNA-Seq and scRNA-Seq have provided immense knowledge of the human islet transcriptional landscape, but each technology has strengths and drawbacks. Despite the prevalence of both approaches and notable studies comparing bulk and single-cell approaches on a common islet preparation (29, 31), this study is, to our knowledge, the first to report direct comparisons of bulk RNA-Seq on FACS-purified human α and β cells and scRNA-Seq on FACS-purified and dispersed cells from the same individuals. We highlight that although sensitivity to low-expression genes was reduced in scRNA-Seq, the detected genes covered a broad range of biological pathways that allowed reconstruction of GO enrichment maps obtained from bulk RNA-Seq (Figure 2, D and F). Further, α and β cells showed very similar expression profiles regardless of cell-type identification method, with neither clustering via transcriptional similarity nor presence of characterized cell-surface proteins showing an apparent bias. This indicates that enrichment methods using cell-surface markers are an appropriate method to investigate a subpopulation of islet cell types.

Lower gene detection in scRNA-Seq compared with bulk was an expected finding given that bulk RNA-Seq generates reads from nearly the entire length of a gene, while the 10x Genomics platform, used in this study, does so only from the 3′ end. Single-cell technologies that capture full-length transcripts (e.g., Smart-Seq2) fare better in direct comparison of gene expression levels (34). Indeed, the smaller working range and lower signal-to-noise ratio were reflected in our scRNA-Seq data. Despite this, detected transcript levels in both data sets converged linearly and were involved in a broad range of similar biological processes, emphasizing the high fidelity of both methods to assess islet cell biology (Figure 2, C–F). To mitigate the differential scale, we also compared the relative transcript abundance in the form of α versus β cell enrichment (Figure 2B). Again, scRNA-Seq was not as sensitive to changes across all transcripts, but those that were detected exhibited very high correlation.

Though it is widely appreciated that numerous TFs act in protein complexes to regulate cellular identity and function, the significance of their heterogenous expression for maintaining identity and function has not been explored. Building on the strength of scRNA-Seq to resolve cell heterogeneity, we explored numerous islet-enriched TFs and found bimodal distribution patterns that suggest the presence of unique combinatorial profiles. In this manuscript, we investigated expression patterns of 3 TFs with known changes in islet cell development and diabetes: α cell–specific *ARX*, β cell–specific *MAFA*, and *MAFB*, which is expressed in both α and β cells and has a unique expression profile compared with rodent islets. Interestingly, other islet-enriched TFs were consistently elevated in *ARX*/*MAFB*-coexpressing α cells and *MAFA*/*MAFB*-coexpressing β cells, supporting the concept of islet-enriched TFs acting in self-regulating networks, and making it likely that combinatorial profiles of other TFs also reveal interesting populations with functional consequences. Larger data sets and network-based approaches considering additional TF combinations should be used to examine more complex expression patterns and how these patterns change in type 1 and 2 diabetes islet cells.

One contribution to bimodal distribution in detection of low-abundance transcripts like TFs in scRNA-Seq is gene dropout, where a gene is detected only in a subset of cells because of low mRNA quantity. Although it is tempting to fully attribute such bimodal distributions to dropout, we showed greater expression of functional genes in one subpopulation (often dual-positive cells), suggesting that dropout is not simply a stochastic event and could instead reflect cell states, cell activity, or a biological process such as transcriptional bursting (52). These findings were replicated in 3 additional scRNA-Seq data sets of human islets generated by various single-cell technologies (18, 28, 29), and all showed trends consistent with the current study. Finally, taking advantage of the Patch-Seq approach from our previous study, we were able to validate increased cellular function reflected by electrophysiological parameters (Figure 6). Thus, while it is not possible with current technologies to prove that cells with undetectable TF expression truly have no expression, these data indicate that such observations are not simply technical in nature and instead are reflective of important underlying human islet biology.

Our data suggest that *ARX*/*MAFB*-coexpressing α cells and *MAFA*/*MAFB*-coexpressing β cells have elevated expression of functional genes compared with cells that express only one or neither factor. Nonetheless, elevated expression for certain genes in single TF-expressing populations (e.g., *MDH2* and *KCNMA1* in *MAFB*-expressing β cells) may provide insight into how these individual TFs act in each cell type. Indeed, a comparison of our data to molecular studies of these TFs in mice or human stem cells revealed numerous similarities. For example, our data demonstrated that *MAFA*/*MAFB-*coexpressing β cells were distinct from populations that expressed only a single TF, which suggests that although these factors are related, they have distinct targets and roles within the β cell. This is consistent with a recent report showing that in mice, MAFB does not compensate for MAFA loss (12). Further, our data highlight *MAFB* as playing a key role in defining both β and α cell identity, in line with a recent report where MAFB deletion in human embryonic stem cells disrupted the differentiation process for both β and α cells (53). Thus, our approach highlights how TF profiles at the single-cell level can be used to predict transcriptional and functional consequences of genetic manipulation, highlighting an immense power for large scRNA-Seq data sets.

Although there were not sufficient cells for robust statistical comparison of all subsets, it is interesting to note that the electrophysiological profile of the cells expressing neither MAFA nor MAFB was similar to those cells expressing only one of the factors, thus suggesting a specific benefit to having combined expression of both factors in adult human β cells that is not apparent with only one of the TFs. These findings have several implications given the unique timing of MAFA and MAFB expression in the human β cell and differ slightly from our transcriptional data that suggested more of a progressive increase, in which the double-negative group showed the lowest expression followed by single TF groups and coexpressing cells had the highest expression of genes related to hormone secretory function. Future investigation with larger functional data sets will be needed to further delineate these interesting findings as well as directly evaluate the role of MAFA, MAFB, and other enriched TFs in human islet cell hormone secretion.

Given the potential inconsistencies between transcript- and protein-level expression in human islets (51), we pursued identification of heterogeneous TF protein expression in human pancreatic tissue. Though there were discrepancies in subpopulation distribution estimated by transcript versus by immunodetection, the presence of all TF combinations in tissue indicates this heterogeneity is not limited to one experimental approach. Differences may also arise from posttranscriptional control of protein levels that would not be apparent at the transcript level. Novel, single-cell multiomic techniques will be required to define the precise correlation between TF mRNA and protein abundance, and these techniques may also help define how the described heterogeneity relates to other forms of β cell heterogeneity that have been previously described or hypothesized (18–22). Heterogeneity within α cell populations has been less studied, but our data indicate it may have an unappreciated role within the islet as well.

There are limitations to the current study that suggest opportunities for future work. First, the dispersion of islet cells required for scRNA-Seq disrupts the microenvironment, which is known to be crucial for coordinated islet function (54, 55). How the α and β cell subpopulations defined in this study function in the islet context is presently unknown — although having all highly functional cells would seem beneficial, some data have suggested that both mature and immature cells are required within an islet for optimal function (56). Importantly, the nature of scRNA-Seq means we cannot discern whether the heterogeneity described here is stable or a snapshot of a dynamic cell state.

In sum, we highlighted the utility of a large, scRNA-Seq data set by uncovering previously unappreciated heterogeneity in combined key islet-enriched TF expression and demonstrated that it has implications for β cell function. Ultimately, defining the key characteristics of highly functional α and β cells will allow not only a greater understanding of pathways governing coordinated hormone secretion but also engineering of cells closely resembling native α or β cell function for cell replacement therapy to treat diabetes.

## Methods

### Human pancreatic islet samples.

Human islet preparations (*n =* 5; see Supplemental Table 1 for donor information) were obtained through partnerships with the Integrated Islet Distribution Program (IIDP, RRID:SCR_014387; http://iidp.coh.org/), Alberta Diabetes Institute (ADI) IsletCore (RRID:SCR_018566; https://www.epicore.ualberta.ca/IsletCore/), and the Human Pancreas Analysis Program (HPAP; RRID:SCR_016202; https://hpap.pmacs.upenn.edu/) of the Human Islet Research Network (HIRN). Assessment of human islet function was performed by islet macroperifusion assay on the day of arrival, as previously described (57). Islets were cultured in CMRL 1066 media (5.5 mM glucose, 10% FBS, 1% penicillin/streptomycin, 2 mM L-glutamine) in 5% CO_2_ at 37^o^C for less than 24 hours prior to beginning studies.

### Cell preparation.

Handpicked pancreatic islets were dispersed by manual pipetting using 0.025% HyClone trypsin (Cytiva/GE Healthcare, SH30042.01) and subsequently quenched with RPMI media containing 20% FBS (MilliporeSigma, TMS-013-B). Cells were washed in the same media twice followed by 1 wash with 0.04% BSA (Thermo Fisher Scientific, AM2616) in 1× PBS without calcium and magnesium (Corning Cellgro, 21-040-CV). Washed cells were immediately counted in a Trypan blue stain–based Cell Countess II Automated Cell Counter (Thermo Fisher Scientific, AMQAX1000). Viability obtained from the cell preparations ranged from 70% to 85%. Cells were resuspended in 0.04% BSA/1× PBS at a density of 630 to 1200 cells/μL in preparation for scRNA-Seq.

*Purification ofαandβcells by FACS*. Human islets from preparations 1 and 2 (Supplemental Table 1) were dispersed and sorted for α and β cells following protocols described previously (17, 27, 58). Briefly, 0.025% trypsin was used to disperse islet cells by manual pipetting and subsequently quenched with RPMI containing 10% FBS. Previously characterized primary and secondary antibodies (25, 27, 59) are listed in Supplemental Table 3, and the gating strategy is shown in Supplemental Figure 2A. Collected α and β cells for scRNA-Seq were washed in 1× PBS with 0.04% BSA and immediately loaded into the 10x Genomics Chromium Controller at 1200 cells/μL based on FACS counts, with single-cell libraries prepared as described below. In parallel, 10,000 α and β cells from islet preparation 2 were stored in RNA extraction buffer to be processed for bulk RNA-Seq as described below.

### Bulk RNA library preparation and sequencing.

RNA was extracted from sorted α and β cells using the Invitrogen RNAqueous-Micro Total RNA Isolation kit (Thermo Fisher Scientific, AM1931). TURBO DNA-free (Ambion) was used to treat any trace DNA contamination. RNA was quantified by Qubit Fluorometer 2.0 and RNA integrity was confirmed (RIN >7) by 2100 Bioanalyzer (Agilent). RNA was amplified using NuGen Ovation RNA amplification kit and sheared to an average size of 200 bp, and then libraries were prepared using the NEBNext DNA library prep kit (New England Biolabs). Final libraries were sequenced on a Novaseq platform (Illumina) using paired-end reads (50 bp) targeting 50 million reads per sample. Raw reads were aligned to human reference genome hg38 using STAR v2.6 (60). Strand NGS 3.4 commercial software was used to import aligned files (.bam) and subsequently check alignment quality, filter reads based on read quality, quantify transcripts, and normalize counts to transcript per million (TPM). Only genes with expression log_2_ (TPM) greater than 1 for bulk data and unique molecular identifiers greater than 1 for 10x Genomics single-cell data were considered for the analysis in Figure 2, B–F. Differential expression analysis between α and β cells was defined as fold change ≥±1, calculated based on *P* value estimated by *z* score calculations (cutoff 0.05) as determined by Benjamini Hochberg FDR correction of 0.05 (61). GO analyses (Figure 2, D and F) and KEGG pathway analysis (Supplemental Figure 2, F and G) were performed using Metascape version 3.5 (49), where the network plot was created from a subset of enriched GO terms clustered by similarity greater than 0.3 (Kappa scores), and a representative term from each of the 20 clusters was hand-selected for labeling. Network was visualized using Cytoscape (62).

For original/source data used in Figure 1, A and C, bulk RNA-Seq data of sorted human islet α cells are available in NCBI’s Gene Expression Omnibus (GEO) under accession number GSE106148 (17) and bulk RNA-Seq data of sorted human islet β cells are available under GSE116559 (27). For Figure 1, B and D and Supplemental Figure 1, A and B, normalized bulk RNA-Seq data (TPM or RPKM) were retrieved from GSE57973 (10) and GSE67543 (26).

### Single-cell library preparation and sequencing.

Sorted or dispersed islet cell samples were loaded in triplicate (approximately 10,000 cells/replicate) on 10x Genomics chromium chips (PN 1000009) to ensure consistent results. Gel bead in emulsion (GEM) generation and barcoding were performed on the 10x Genomics Chromium Controller according to the manufacturer’s instructions (10x Genomics Single Cell 3′ Library and Gel Bead kit v2, 220104). Immediately after GEMs were generated, samples were transferred to a 0.2 mL TempAssure PCR 8-tube strip (USA Scientific, 14024700), capped, and placed into a thermocycler (Bio-Rad T100 Thermal Cycler) for reverse transcription. After incubation, the GEMs were broken, and pooled cDNA proceeded to cleanup using Silane magnetic beads (10x Genomics, 2000048) to remove leftover reagents. cDNA was then amplified through 10 cycles of PCR and cleaned using SPRIselect beads (Beckman Coulter, B23318). Resulting cDNA (average 45 ng/replicate) was checked for quality by Qubit dsDNA HS Assay Kit (Thermo Fisher Scientific, Q32854) and Agilent Bioanalyzer High Sensitivity Kit (5067-4626). Final libraries were constructed according to the manufacturer’s instructions and underwent 14 cycles of PCR amplification after sample index addition, yielding approximately 953 ng and average library size of 486 bp. Final libraries were sequenced with a Novaseq sequencer (Illumina) using paired-end reads (100 bp) to average depth of approximately 146,000 reads per cell.

### scRNA-Seq alignment, preprocessing, and quality control.

Alignment to reference transcriptome (GRCh38-1.2; gene annotation provided by 10x Genomics) and unique molecular identifier–based gene expression quantification was obtained following the Cell Ranger analysis pipeline (v2.1). The “Aggr” function was used to aggregate transcript counts and normalize read depth across 5 islet preparations and their technical replicates, producing 1 single gene-cell (feature-barcode) matrix. In Figure 2, G–I, the CellRanger “Aggr” function was applied to 2 islet preparations, including the samples that were FACS sorted. Further data preprocessing and clustering were performed using Seurat version 3.1 (50).

Cells with 200 to 4000 detected genes and less than 10% mitochondrial gene expression were retained, and only genes expressed in 3 or more cells were considered for further analysis. Gene expression was normalized for each cell by library size and log-transformed using a size factor of 10,000 molecules per cell. For feature selection, 2000 highly variable genes were selected using function “FindVariableFeatures.” The data were further centered and scaled to zero mean and unit variance implemented in the “ScaleData” function using parameter “vars.to.regress” to regress out mitochondrial gene expression. Cells coexpressing the insulin (*INS*) and glucagon (*GCG*) genes above log expression of 6.5 and 5, respectively, as well as cells expressing *INS* or *GCG* in addition to any other cell-type gene marker, were removed as doublets (see Supplemental Table 2 for cell-type markers used). Transcript counts from lysed cells (ambient mRNA/background RNA) were estimated and genes identified from empty droplets (droplets without cells) using DropletUtils package (63). Using the raw gene-barcode matrix (Cell Ranger v3.1), a unique molecular identifier count threshold of 100 and below was used to identify ambient transcripts. Approximately 200 genes were identified as ambient genes, and their expression level was noted to remove from the original gene barcode matrix in order to account for transcript stemming from lysed cells. PCA was performed using previously determined 2000 high variable genes as input. An elbow plot, which ranks the principal components (PCs) based on percentage variance per PC, was considered to determine the number of PCs to use for downstream graph-based clustering. “FindNeighbors” and “FindClusters” functions were used with 20 PCs as input for cluster generation and resolution at 0.6. Cells from dispersed WIs, FACS-α, and FACS-β samples (*n =* 27,614 in total) were analyzed by graph-based unsupervised clustering applying Louvain algorithm (50, 64) and visualized using uniform manifold approximation and projection (UMAP; ref. 65), and α and β cells were annotated with markers (Supplemental Table 2) overlaid to unsupervised clusters. Analysis of cell-cycle state (Supplemental Figure 3F) was performed using the standard gene list included in the Seurat R package (66).

### Immunohistochemical analysis.

Lightly paraformaldehyde-fixed human pancreatic tissue cryosections from *n =* 3 donors (age range 20–55 years) were prepared for immunohistochemistry and stained as described previously (17, 27, 58). Primary and secondary antibodies and their dilutions are listed in Supplemental Table 3; donor information is supplied in Supplemental Table 4. Images were acquired at 20× with 2× digital zoom using a FV3000 confocal laser scanning microscope (Olympus) and processed using HALO software (Indica Labs) with a cytonuclear algorithm (HighPlex FL v3.2.1) to set an intensity threshold (“hi/lo”) for each marker.

### Analysis of previously published scRNA-Seq data sets.

Raw gene count matrices were extracted from existing scRNA-Seq data sets (18, 28, 29) and further analyzed using the R package Seurat version 3.1 as described above.

### Single-cell electrophysiology and gene expression.

Patch-Seq was performed as described previously in Camunas et al. (18).

### Data availability.

GEO for sequencing data sets (GSE183568). Single-cell data set visualization can be found at https://powersbrissovalab.shinyapps.io/scRNAseq-Islets/

### Statistics.

Specific statistical tests used for each data set are described in the figure legends and text where appropriate. All Student’s *t* tests were 2-tailed, with a *P* value less than 0.05 considered significant. In the case of 2-way ANOVA (Supplemental Figures 4 and 6), a *P* value less than 0.05 was considered significant and was followed by Tukey’s multiple-comparison test, also at a threshold of *P* less than 0.05. Pearson’s correlation (Figure 2, C and E and Supplemental Figure 2, D and E) was performed using the ggpubr package (http://rpkgs.datanovia.com/ggpubr/). GO enrichment and network visualization (Figure 2, D and F) were performed using Metascape 3.5, where *P* values are calculated based on the accumulative hypergeometric distribution (67). All other statistical analyses were performed using GraphPad Prism software.

### Study approval.

The Vanderbilt University IRB does not consider deidentified human pancreatic specimens to qualify as human subject research. This study used data from the Organ Procurement and Transplantation Network (OPTN) that were in part compiled from the data hub accessible to IIDP-affiliated investigators through the IIDP portal (https://iidp.coh.org/secure/isletavail). The OPTN data system includes data on all donors, waitlisted candidates, and transplant recipients in the US submitted by the members of the OPTN. The Health Resources and Services Administration of the US Department of Health and Human Services provides oversight to the activities of the OPTN contractor. The data reported here have been supplied by United Network for Organ Sharing as the contractor for the OPTN. The interpretation and reporting of these data are the responsibility of the authors and in no way should be seen as an official policy or interpretation of the OPTN or the US government.

## Author contributions

SS, DCS, JTW, MB, RS, and ACP contributed to conceptualization; SS, DCS, JTW, JCS, and XQD contributed to methodology; SS, DCS, JTW, XQD, RH, RA, GP, and RB performed the investigation; SS, JCS, and JPC performed the formal analysis; SS, DCS, and JTW wrote the original draft; all authors reviewed and edited the manuscript; PEM, SEL, ACP, and MB acquired funding; NP, JPC, SCJP, PEM, SEL, ACP, and MB supervised the study.

## Supplementary Material

Supplemental data

## Version 1 08/24/2021

In-Press Preview

## Version 2 09/22/2021

Electronic publication

## References

[1] Noguchi GM, Huising MO (2019). Integrating the inputs that shape pancreatic islet hormone release. Nat Metab.

[2] Chen C (2017). Human beta cell mass and function in diabetes: recent advances in knowledge and technologies to understand disease pathogenesis. Mol Metab.

[3] Cnop M (2005). Mechanisms of pancreatic β-cell death in type 1 and type 2 diabetes: many differences, few similarities. Diabetes.

[4] Halban PA (2014). β-cell failure in type 2 diabetes: postulated mechanisms and prospects for prevention and treatment. Diabetes Care.

[5] Unger RH, Cherrington AD (2012). Glucagonocentric restructuring of diabetes: a pathophysiologic and therapeutic makeover. J Clin Invest.

[6] Pan FC, Wright C (2011). Pancreas organogenesis: from bud to plexus to gland. Dev Dyn.

[7] Jennings RE (2015). Human pancreas development. Development.

[8] Zhu Z (2016). Genome editing of lineage determinants in human pluripotent stem cells reveals mechanisms of pancreatic development and diabetes. Cell Stem Cell.

[9] Thompson P, Bhushan A (2017). β cells led astray by transcription factors and the company they keep. J Clin Invest.

[10] Arda HE (2016). Age-dependent pancreatic gene regulation reveals mechanisms governing human β cell function. Cell Metab.

[11] Dai C (2012). Islet-enriched gene expression and glucose-induced insulin secretion in human and mouse islets. Diabetologia.

[12] Cyphert HA (2018). Examining how the MAFB transcription factor affects islet β-cell function postnatally. Diabetes.

[13] Hang Y (2014). The MafA transcription factor becomes essential to islet β-cells soon after birth. Diabetes.

[14] Guo S (2013). Inactivation of specific β cell transcription factors in type 2 diabetes. J Clin Invest.

[15] Dai C (2016). Stress-impaired transcription factor expression and insulin secretion in transplanted human islets. J Clin Invest.

[16] Talchai C (2012). Pancreatic β cell dedifferentiation as a mechanism of diabetic β cell failure. Cell.

[17] Brissova M (2018). α cell function and gene expression are compromised in type 1 diabetes. Cell Rep.

[18] Camunas-Soler J (2020). Patch-seq links single-cell transcriptomes to human islet dysfunction in diabetes. Cell Metab.

[19] Dorrell C (2016). Human islets contain four distinct subtypes of β cells. Nat Commun.

[20] Wang YJ (2016). Single-cell mass cytometry analysis of the human endocrine pancreas. Cell Metab.

[21] Thompson PJ (2019). Targeted elimination of senescent beta cells prevents type 1 diabetes. Cell Metab.

[22] Meulen T van der, Huising MO (2014). Maturation of stem cell-derived beta-cells guided by the expression of urocortin 3. Rev Diabet Stud.

[23] Fadista J (2014). Global genomic and transcriptomic analysis of human pancreatic islets reveals novel genes influencing glucose metabolism. Proc Natl Acad Sci U S A.

[24] Eizirik DL (2012). The human pancreatic islet transcriptome: expression of candidate genes for type 1 diabetes and the impact of pro-inflammatory cytokines. PLoS Genet.

[25] Dorrell C (2011). Transcriptomes of the major human pancreatic cell types. Diabetologia.

[26] Blodgett DM (2015). Novel observations from next-generation RNA sequencing of highly purified human adult and fetal islet cell subsets. Diabetes.

[27] Saunders DC (2019). Ectonucleoside triphosphate diphosphohydrolase-3 antibody targets adult human pancreatic β cells for in vitro and in vivo analysis. Cell Metab.

[28] Baron M (2016). A single-cell transcriptomic map of the human and mouse pancreas reveals inter- and intra-cell population structure. Cell Syst.

[29] Segerstolpe Å (2016). Single-cell transcriptome profiling of human pancreatic islets in health and type 2 diabetes. Cell Metab.

[30] Xin Y (2016). RNA sequencing of single human islet cells reveals type 2 diabetes genes. Cell Metab.

[31] Lawlor N (2017). Single-cell transcriptomes identify human islet cell signatures and reveal cell-type-specific expression changes in type 2 diabetes. Genome Res.

[32] Fang Z (2019). Single-cell heterogeneity analysis and CRISPR screen identify key β-cell-specific disease genes. Cell Rep.

[33] Wang YJ, Kaestner KH (2019). Single-cell RNA-seq of the pancreatic islets — a promise not yet fulfilled?. Cell Metab.

[34] Mawla AM, Huising MO (2019). Navigating the depths and avoiding the shallows of pancreatic islet cell transcriptomes. Diabetes.

[35] Itoh M (2010). Partial loss of pancreas endocrine and exocrine cells of human ARX-null mutation: consideration of pancreas differentiation. Differentiation.

[36] Gosmain Y (2011). Glucagon gene expression in the endocrine pancreas: the role of the transcription factor Pax6 in α-cell differentiation, glucagon biosynthesis and secretion. Diabetes Obes Metab.

[37] Courtney M (2013). The inactivation of Arx in pancreatic α-cells triggers their neogenesis and conversion into functional β-like cells. PLoS Genet.

[38] Wang H (2007). MAFA controls genes implicated in insulin biosynthesis and secretion. Diabetologia.

[39] Bonnavion R (2013). Both PAX4 and MAFA are expressed in a substantial proportion of normal human pancreatic alpha cells and deregulated in patients with type 2 diabetes. PLoS One.

[40] Liu W (2019). Abnormal regulation of glucagon secretion by human islet alpha cells in the absence of beta cells. EBioMedicine.

[41] Matsuoka T (2004). The MafA transcription factor appears to be responsible for tissue-specific expression of insulin. Proc Natl Acad Sci U S A.

[42] Matsuoka T (2007). MafA regulates expression of genes important to islet beta-cell function. Mol Endocrinol.

[43] Artner I (2010). MafA and MafB regulate genes critical to beta-cells in a unique temporal manner. Diabetes.

[44] Otonkoski T (1988). Maturation of insulin response to glucose during human fetal and neonatal development. Studies with perifusion of pancreatic isletlike cell clusters. Diabetes.

[45] Henquin JC, Nenquin M (2016). Dynamics and regulation of insulin secretion in pancreatic islets from normal young children. PLoS One.

[46] Helman A (2020). A nutrient-sensing transition at birth triggers glucose-responsive insulin secretion. Cell Metab.

[47] Matsuoka T (2003). Members of the large Maf transcription family regulate insulin gene transcription in islet beta cells. Mol Cell Biol.

[48] Dorrell C (2008). Isolation of major pancreatic cell types and long-term culture-initiating cells using novel human surface markers. Stem Cell Res.

[49] Zhou Y (2019). Metascape provides a biologist-oriented resource for the analysis of systems-level datasets. Nat Commun.

[50] Butler A (2018). Integrating single-cell transcriptomic data across different conditions, technologies, and species. Nat Biotechnol.

[51] Wortham M (2018). Integrated in vivo quantitative proteomics and nutrient tracing reveals age-related metabolic rewiring of pancreatic β cell function. Cell Reports.

[52] Kharchenko PV (2014). Bayesian approach to single-cell differential expression analysis. Nat Methods.

[53] Russell R (2020). Loss of the transcription factor MAFB limits β-cell derivation from human PSCs. Nat Commun.

[54] Elliott AD (2015). Somatostatin and insulin mediate glucose-inhibited glucagon secretion in the pancreatic α-cell by lowering cAMP. Am J Physiol Endocrinol Metab.

[55] Capozzi ME (2019). β-Cell tone is defined by proglucagon peptides through cyclic AMP signaling. JCI Insight.

[56] Nasteska D (2021). PDX1LOW MAFALOW β-cells contribute to islet function and insulin release. Nat Commun.

[57] Kayton NS (2015). Human islet preparations distributed for research exhibit a variety of insulin-secretory profiles. Am J Physiol Endocrinol Metab.

[58] Haliyur R (2018). Human islets expressing HNF1A variant have defective β cell transcriptional regulatory networks. J Clin Invest.

[59] Bramswig NC (2013). Epigenomic plasticity enables human pancreatic α to β cell reprogramming. J Clin Invest.

[60] Dobin A (2013). STAR: ultrafast universal RNA-seq aligner. Bioinformatics.

[61] Benjamini Y, Hochberg Y (1995). Controlling the false discovery rate: a practical and powerful approach to multiple testing. J Royal Statistical Soc Ser B Methodol.

[62] Shannon P (2003). Cytoscape: a software environment for integrated models of biomolecular interaction networks. Genome Res.

[63] Lun ATL (2019). EmptyDrops: distinguishing cells from empty droplets in droplet-based single-cell RNA sequencing data. Genome Biol.

[64] Stuart T (2019). Comprehensive integration of single-cell data. Cell.

[65] McInnes L (2018). UMAP: Uniform Manifold Approximation and Projection. J Open Source Softw.

[66] Tirosh I (2016). Dissecting the multicellular ecosystem of metastatic melanoma by single-cell RNA-seq. Science.

[67] Zar JH. *Biostatistical Analysis 4th ed*. NJ Prentice Hall; 1999.

