# Supplementary material for: Combinatorial transcription factor profiles predict mature and functional human islet α and β cells
Source: JCI Insight. 2021 Sep 22;6(18):e151621. doi: 10.1172/jci.insight.151621 (PMC8492318; doi:10.1172/jci.insight.151621)
Supplement: Supplemental data [file jciinsight-6-151621-s114.pdf]

Combinatorial transcription factor profiles predict mature and functional human islet α and β cells

Shristi Shrestha*, Diane C. Saunders*, John T. Walker*, Joan Camunas-Soler, Xiao-Qing Dai, Rachana Haliyur, Radhika Aramandla, Greg Poffenberger, Nripesh Prasad, Rita Bottino, Roland Stein, Jean-Philippe Cartailler, Stephen C. J. Parker, Patrick E. MacDonald, Shawn E. Levy, Alvin C. Powers[#], Marcela Brissova[#]

* These authors contributed equally, [#] Corresponding authors

Supplemental Information

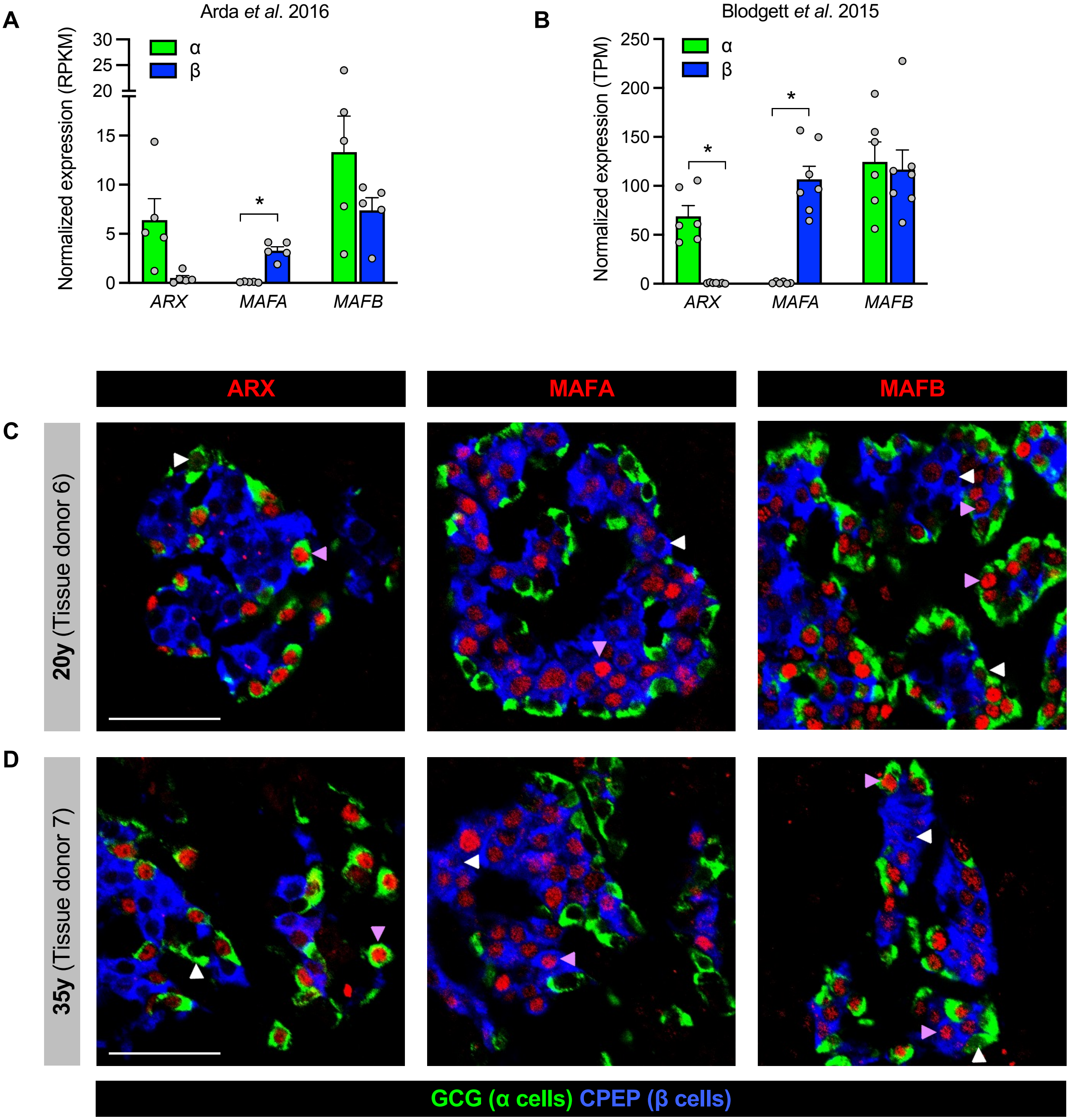

Supplemental Figure 1. ARX is expressed specifically in human α cells and MAFA in β cells, while MAFB is expressed in both α and β cells. (A–B) Normalized expression of *ARX*, *MAFA*, and *MAFB* in previously published bulk RNA-sequencing (RNA-seq) datasets Arda *et al*. 2016 (10) (**A**) and Blodgett *et al*. 2015 (26) (**B**) from α cells (green) and β cells (blue). Bars in both panels show mean + SEM; symbols represent individual donors. Asterisks indicate significantly different (adjusted p-value <0.05) fold change (α vs. β). See also **Figure 1B**. **(C–D)** Immunohistochemical staining of pancreatic sections from nondiabetic adults (**Table S4**), showing specificity of ARX, MAFA, and MAFB (red) in α cells (GCG; green) and β cells (CPEP; blue). Arrowheads indicate cells negative (white) or positive (purple) for transcription factors; scale bars, 50 μm. See also **Figure 1C**.

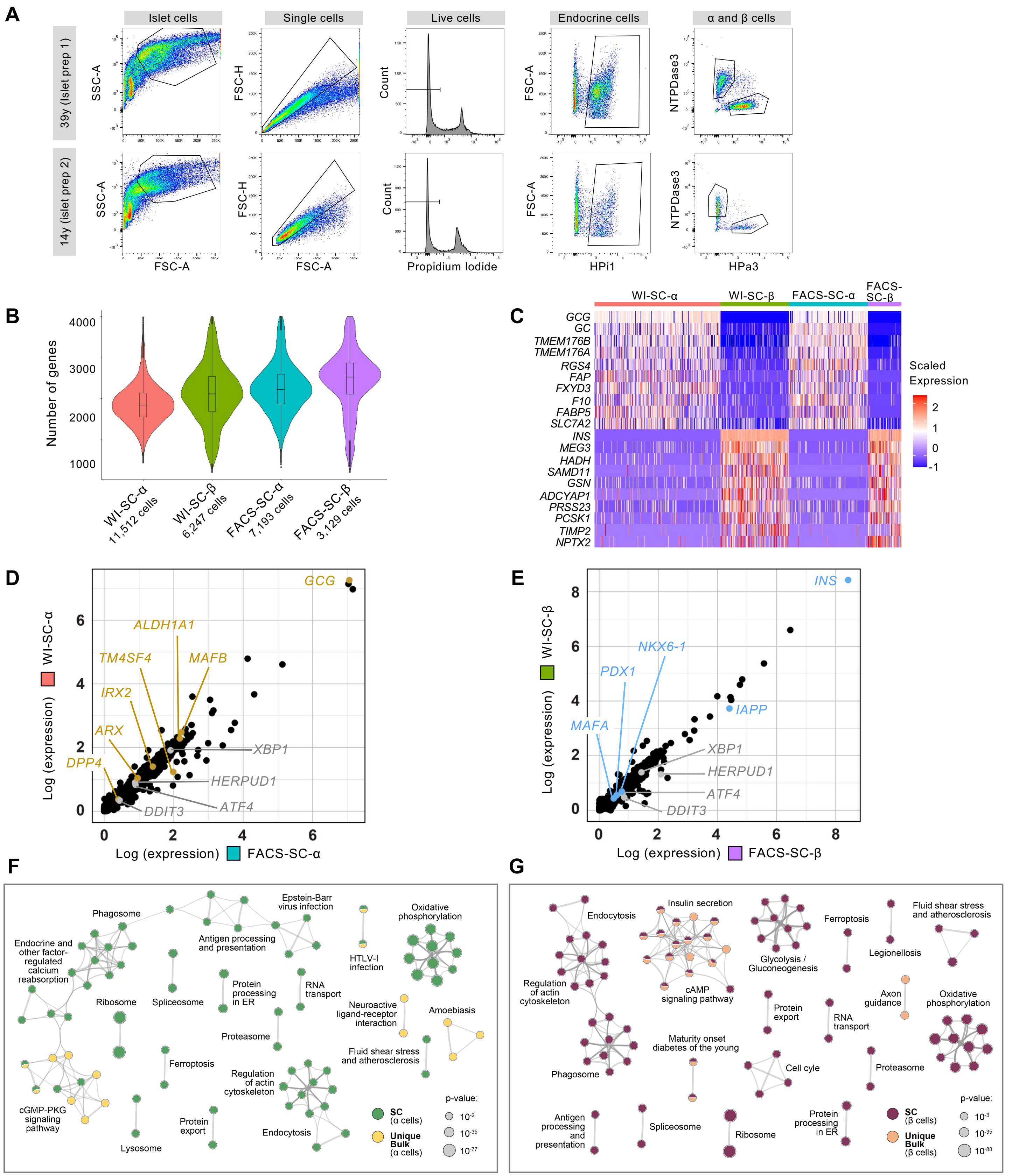

Supplemental Figure 2. Purified α and β cells analyzed by cell surface markers and those identified by unsupervised clustering. (A) Gating strategy for sorted α and β cells identified by cell surface markers. Cell debris were excluded by forward scatter (FSC) and side scatter (SSC), single cells were identified by voltage pulse geometry (FSC-A v. FSC-H), and non-viable cells were excluded using propidium iodide (PI). Endocrine cell subpopulations were then isolated based on positivity for HPi1 (pan-endocrine marker) and additional positivity for HPa3 (α cells) or NTPDase3 (β cells). Antibody information can be found in **Table S3**. **(B)** Number of genes detected was similar among α and β cells identified by cell surface markers and unsupervised clustering. See **Figure 2A** for schematic of experimental design. **(C)** Heatmap depicts expression of genes contributing to variability in PCA of **Figure 2G. (D–E)** Comparison of average log expression of genes across cells identified by unsupervised clustering or cell surface markers for α (**D**) and β cells (**E**). Genes highlighted are α cell-enriched (yellow), β cell-enriched (blue), or selected markers of cell stress (grey). **(F–G)** KEGG enrichment performed on genes common between scRNA-seq and bulk RNA-seq as well as on the 1,000 most highly expressed genes unique to bulk RNA-seq (F, α cells; G, β cells) using Metascape.
See also **Figure 2D** and **2F**.

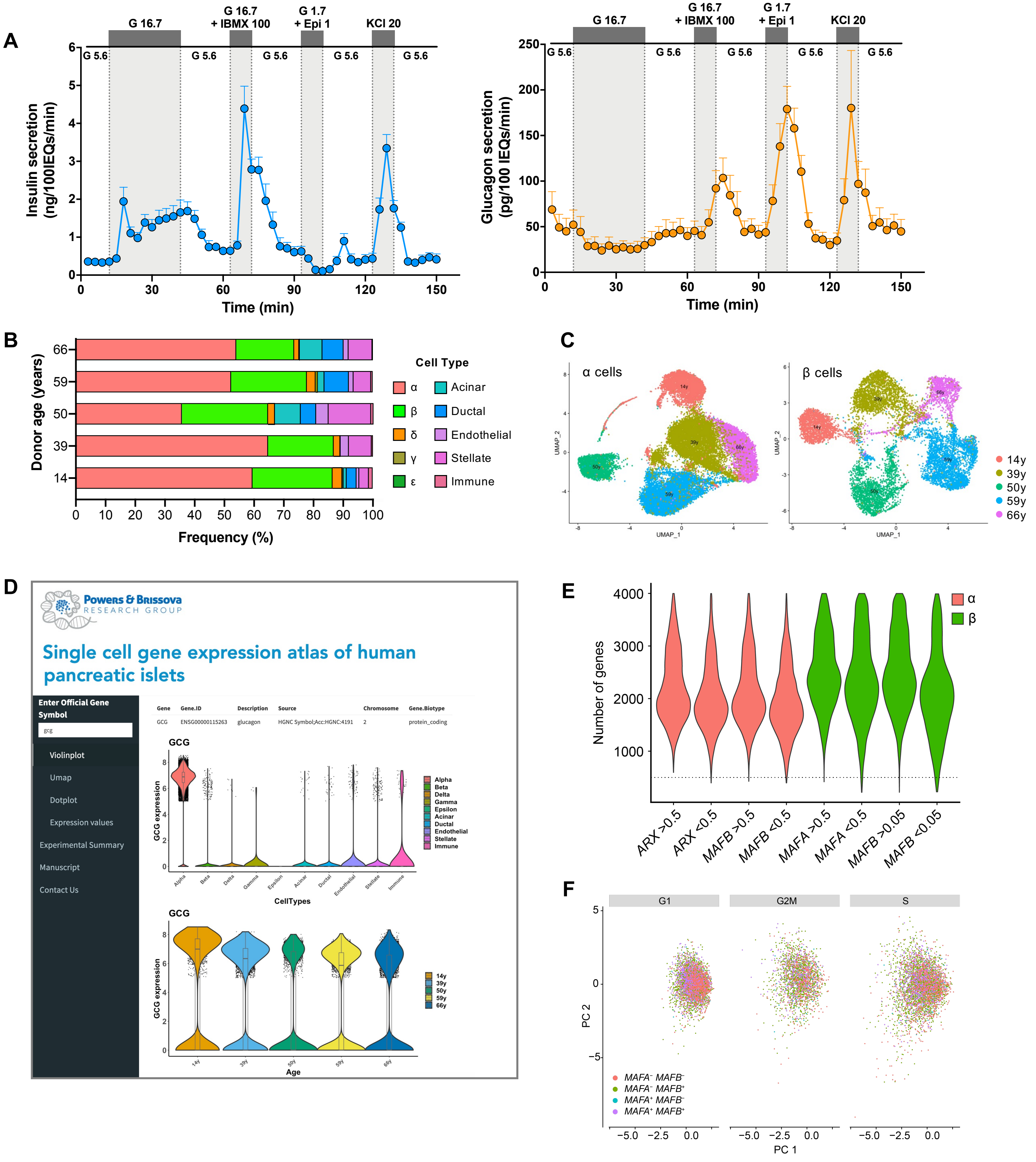

Supplemental Figure 3. Detailed characterization of endocrine cells from five nondiabetic human islet donors. (A) Insulin and glucagon secretion were assessed in islets isolated from n=5 donors (age range 14–66 years) stimulated with 5.6 mM glucose (G 5.6), 16.7 mM glucose (G 16.7), 16.7 mM glucose + 100 mM isobutylmethylxanthine (IBMX) (G 16.7 + IBMX 100), 1.7 mM glucose + 1 mM epinephrine (G 1.7 + Epi 1), and 20 mM potassium chloride (KCl 20). Insulin and glucagon secretion is normalized to overall islet cell volume (expressed as islet equivalents; IEQs). **(B)** Bar graph illustrating cell type distribution within each islet preparation as per cell types annotated in **Figure 3A**. **(C)** UMAP of only α or β cells, showing clustering by islet preparation. See also **Figures 4A** and **5A**. **(D)** User-friendly web portal application for searching and viewing pancreatic cell types and their gene expression; available at https://powersbrissovalab.shinyapps.io/scRNAseq-Islets/. **(E)** Number of genes detected in cells expressing low (ln[UMI per 10,000 +1] <0.5) or high (ln[UMI per 10,000 +1] >0.5) levels of transcription factors *ARX* and *MAFB* in α cells (pink) and *MAFA* and *MAFB* in β cells (green); dotted line represents quality threshold of 500 genes. **(F)** PCA showing cell cycle genes among the four *MAFA*/*MAFB* β cell populations.

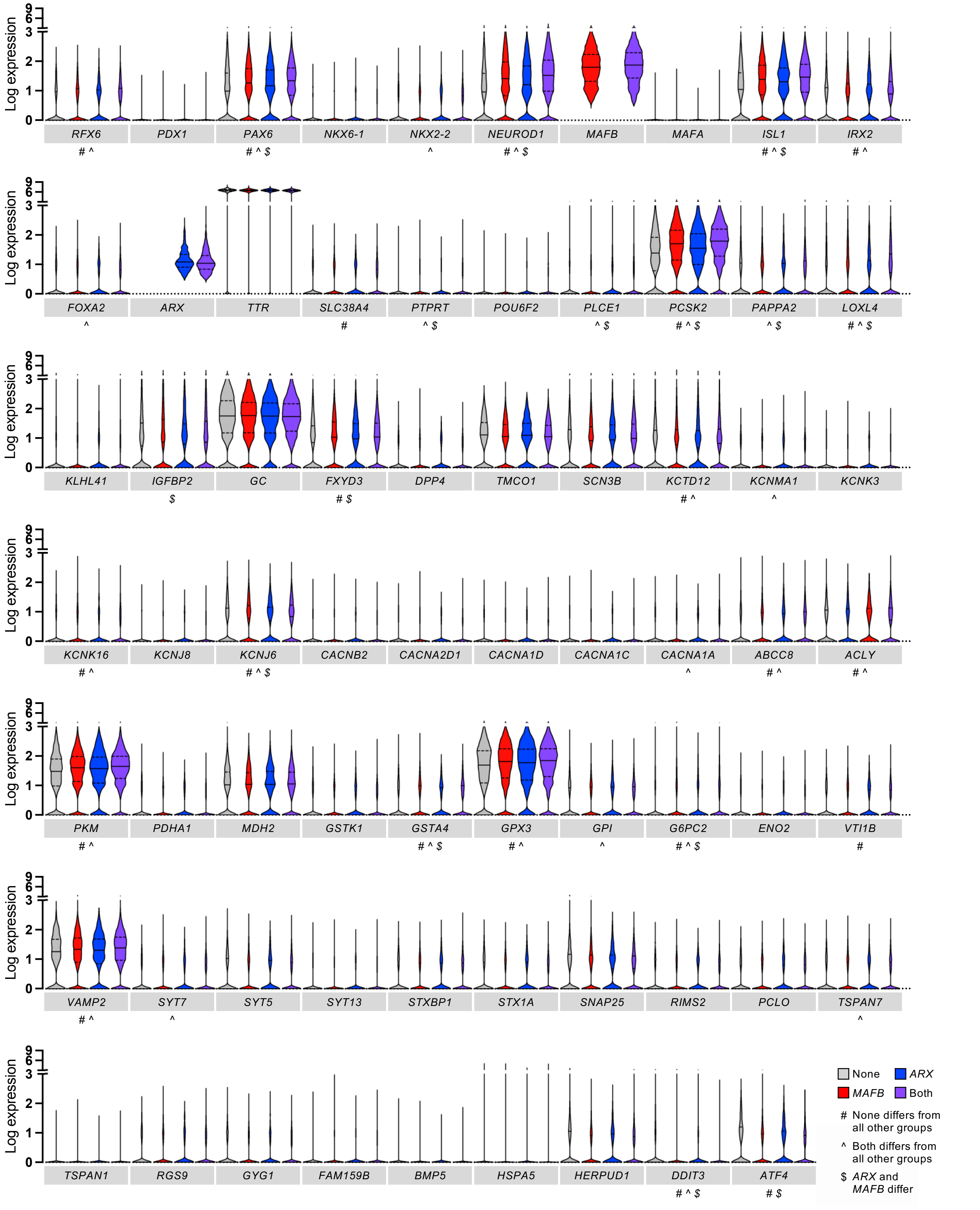

Supplemental Figure 4. Raw expression values for transcription factor, α cell-enriched, ion channel, glucose metabolism, vesicle trafficking, exocytosis, and stress genes in *ARX*/*MAFB* populations. Violin plots depict gene expression in α cell populations lacking *ARX* and *MAFB* (grey) and those expressing *MAFB* only (red), *ARX* only (blue), or co-expressing *both MAFB* and *ARX* (purple); n=24,248 total α cells. Data corresponds to dot plot in **Figure 4C**. Symbols underneath gene names indicate significance ($p<0.05$) from Tukey's multiple comparisons test following 2-way ANOVA.

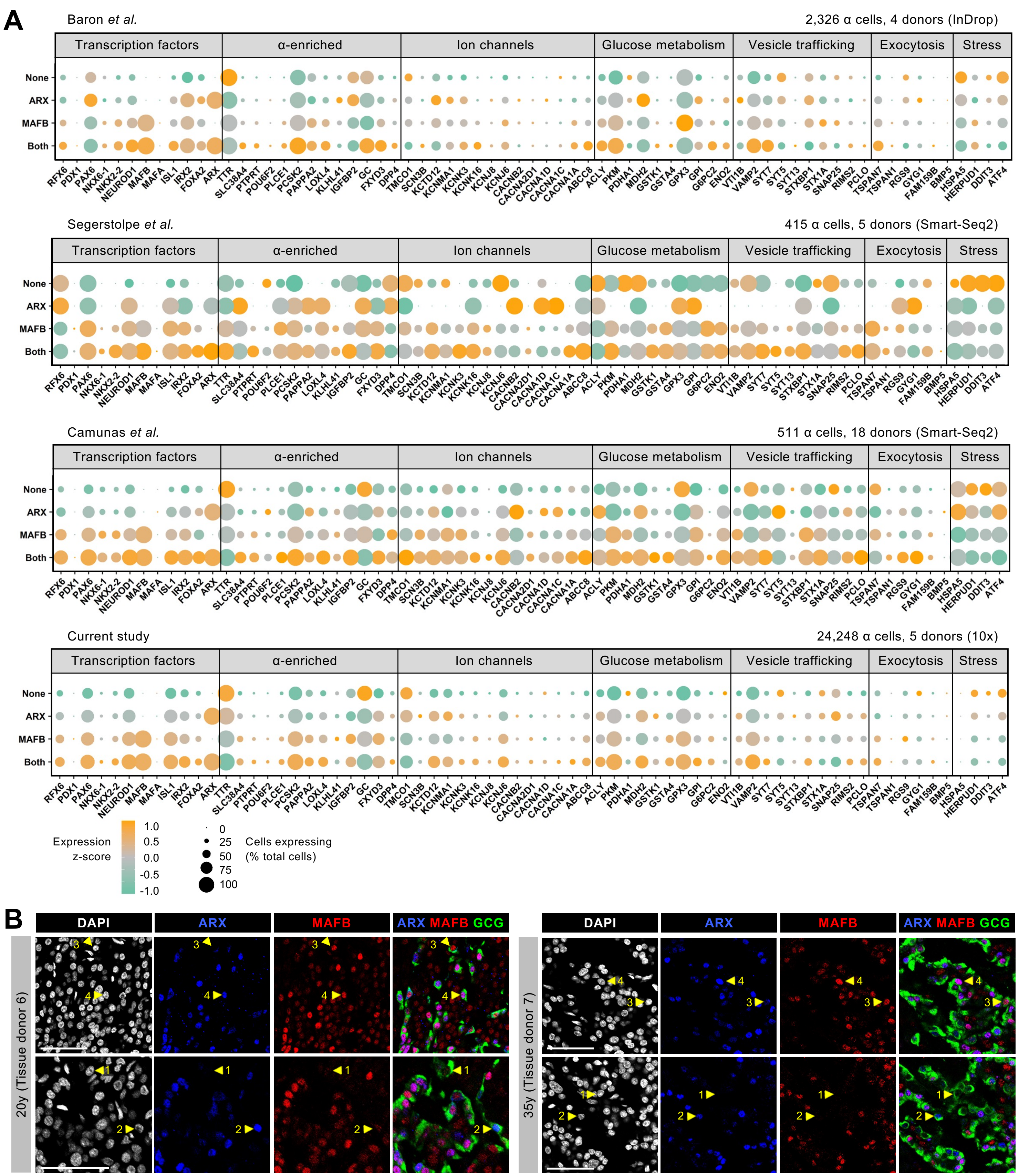

Supplemental Figure 5. Validation of α cell populations based on *ARX* and *MAFB* expression, as determined by previous scRNA-seq studies. (A) Dot plots showing the expression patterns of selected genes related to cell identify, ion flux, glucose metabolism, vesicle trafficking, and exocytotic machinery. α cell populations (rows) are identified by expression of neither *ARX* nor *MAFB* (None), *ARX* only (ARX), *MAFB* only (MAFB), and co-expression of *ARX* and *MAFB* (Both). Dot size indicates the percentage of cells with detectable transcripts; color indicates gene's average scaled expression. Headers list study details from previously published datasets (Segerstolpe *et al.*, 2016 [29]; Baron *et al.*, 2016 [28]; Camunas-Soler *et al.*, 2020 [18]) and final dot plot is as shown in **Figure 4C** for comparison. **(B)** Immunohistochemical staining of ARX (blue) and MAFB (red) in glucagon (GCG)-expressing α cells (green) of two nondiabetic adults (**Table S4**). Numbered arrowheads indicate the presence of α cell populations: 1, ARX^{lo} MAFB^{lo}; 2, ARX^{hi} MAFB^{lo}; 3, ARX^{lo} MAFB^{hi}; 4, ARX^{hi} MAFB^{hi}. See also **Figure 4E**.

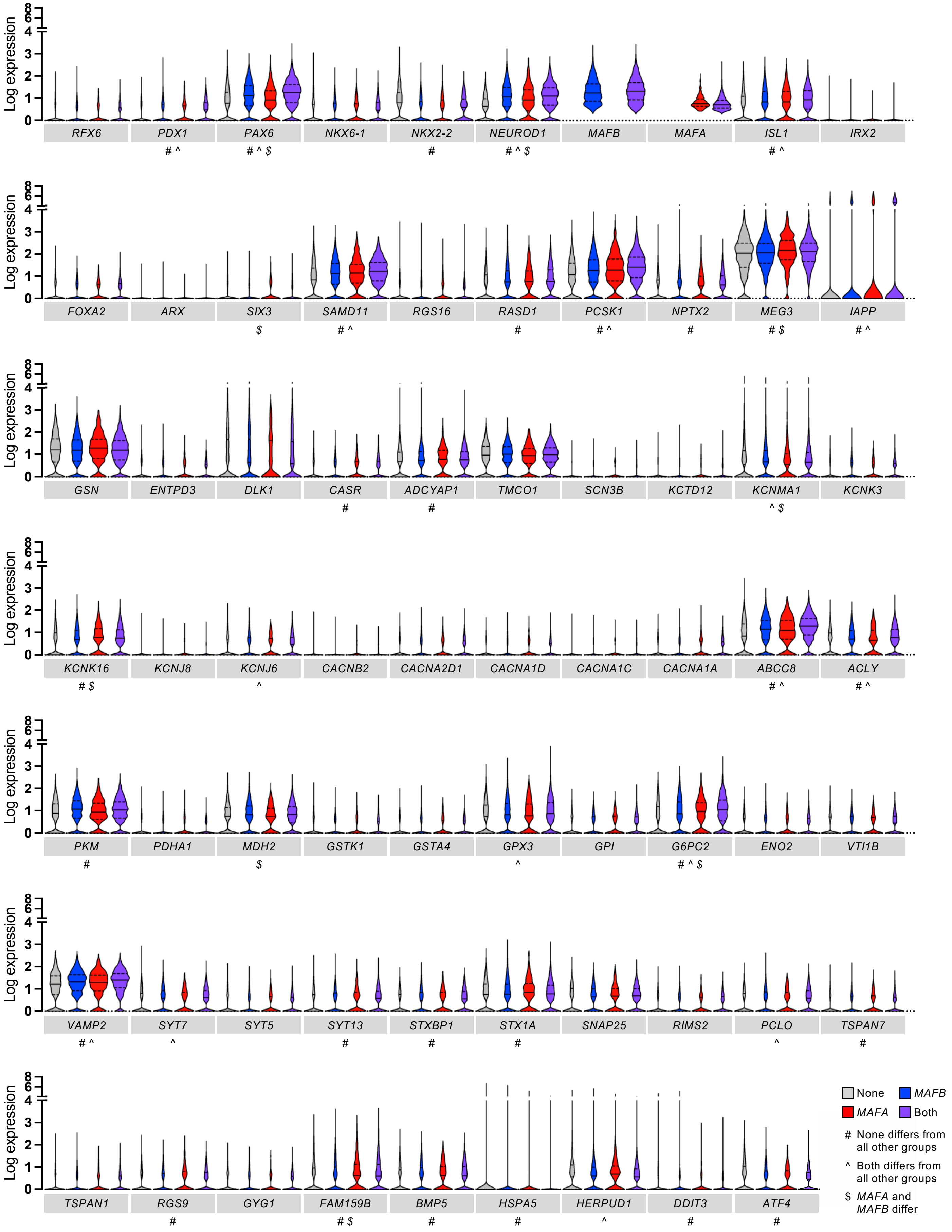

Supplemental Figure 6. Raw expression values for transcription factor, β-enriched, ion channel, glucose metabolism, vesicle trafficking, exocytosis, and stress genes in *MAFA*/*MAFB* populations. Violin plots depict gene expression in β cell populations (n=11,034 total β cells) lacking *MAFA* and *MAFB* (grey) and those expressing *MAFA* only (red), *MAFB only* (blue), or co-expressing *both MAFA* and *MAFB* (purple); n=11,034 total β cells. Data corresponds to dot plot in **Figure 5C**. Symbols underneath gene names indicate significance (p<0.05) from Tukey's multiple comparisons test following 2-way ANOVA.

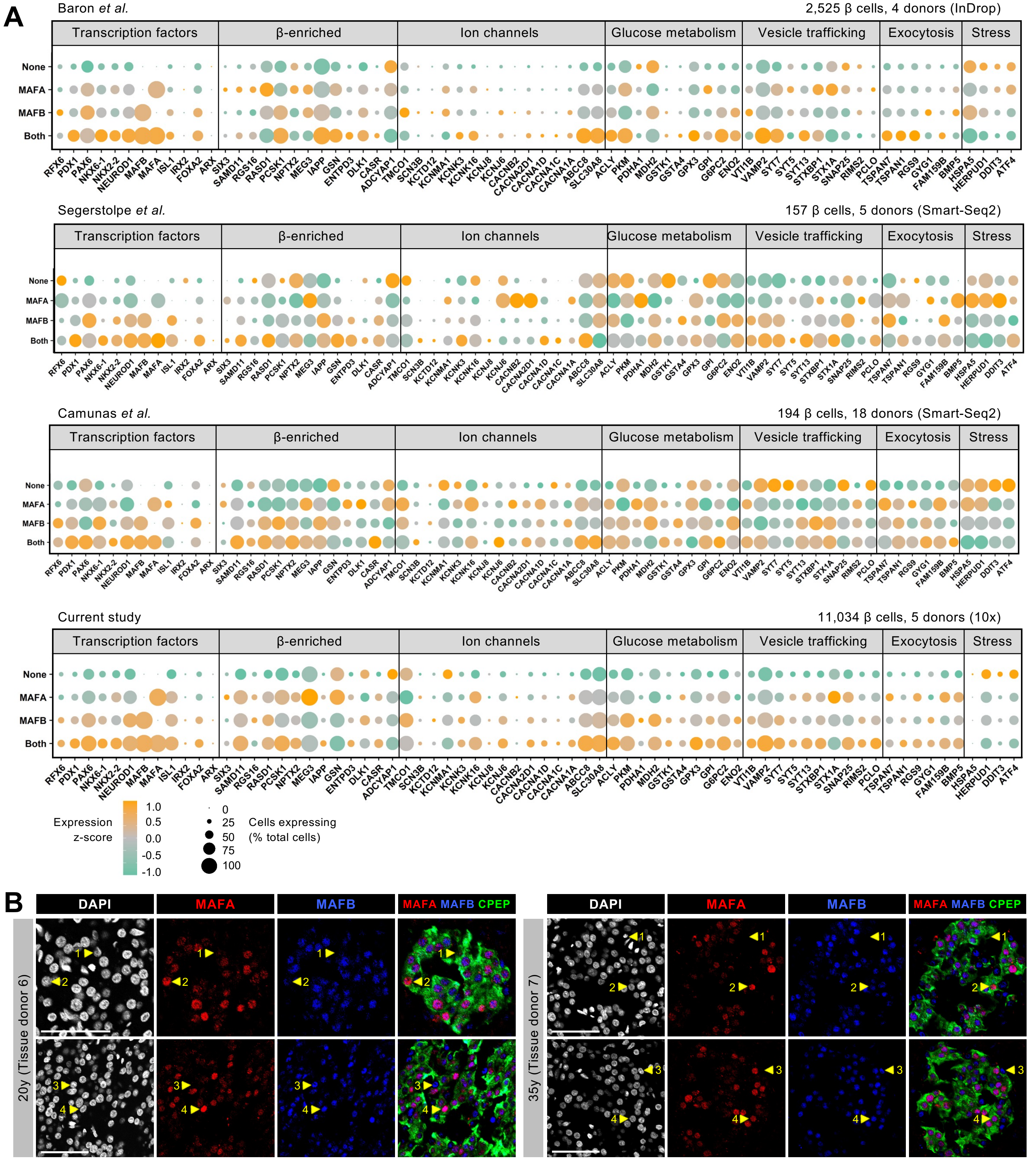

Supplemental Figure 7. Validation of β cell populations based on *MAFA* and *MAFB* expression, as determined by previous scRNA-seq studies. (A) Dot plots showing the expression patterns of selected genes related to cell identify, ion flux, glucose metabolism, vesicle trafficking, and exocytotic machinery. β cell populations (rows) are identified by expression of neither *MAFA* nor *MAFB* (None), *MAFA* only (MAFA), *MAFB* only (MAFB), and co-expression of *MAFA* and *MAFB* (Both). Dot size indicates the percentage of cells with detectable transcripts; color indicates gene's average scaled expression. Headers list study details from previously published datasets (Segerstolpe *et al.*, 2016 [29]; Baron *et al.*, 2016 [28]; Camunas-Soler *et al.*, 2020 [18]) and final dot plot is as shown in **Figure 5C** for comparison. **(B)** Immunohistochemical staining of MAFA (red) and MAFB (blue) in C-peptide (CPEP)-expressing β cells (green) of two nondiabetic adults (**Table S4**). Numbered arrowheads indicate the presence of β cell populations: 1, MAFA^{lo} MAFB^{lo}; 2, MAFA^{hi} MAFB^{lo}; 3, MAFA^{lo} MAFB^{hi}; 4, MAFA^{hi} MAFB^{hi}. See also **Figure 5E**.

Table S1. Human Islet Donor Information

Checklist for Reporting Human Islet Preparations Used in Research
Adapted from Hart NJ, Powers AC (2018) Progress, challenges, and suggestions for using human islets to understand islet biology and human diabetes. Diabetologia https://doi.org/10.1007/s00125-018-4772-2.

Islet preparation	1	2	3	4	5
Unique identifier	DON184	DON185	RRID: SAMN08768781	RRID: SAMN08768783	R232
Donor age (years)	14	39	50	59	66
Donor sex	Male	Female	Male	Female	Female
Donor BMI (kg/m^2)	24.13	34.76	22.4	32.3	18.5
Donor HbA1c (ng/mL)	5.4	4.7	N/A	N/A	6.1
Origin/source of islets	OPO	OPO	IIDP	IIDP	ADI
Islet isolation center	University of Pennsylvania	University of Pennsylvania	University of Pennsylvania	University of Wisconsin	University of Alberta
Donor history of diabetes? Yes/No	No	No	No	No	No
Donor cause of death	Anoxia/ Cardiovascular	Anoxia/ Drug intoxication	Cerebrovascular/ Stroke	Cerebrovascular/ Stroke	Cerebrovascular/ Stroke
Glucose-stimulated insulin secretion or other functional measurement	Perifusion	Perifusion	Perifusion	Perifusion	Perifusion
Handpicked to purity? Yes/No	Yes	Yes	Yes	Yes	Yes
Warm ischaemia time (hours)	N/A	N/A	No	No	N/A
Cold ischaemia time (hours)	12	8.5	9.9	N/A	N/A
Estimated purity (%)	75	95	95	95	90
Total culture time (hours)	36	96	19	44	144

ADI, Alberta Diabetes Institute; IIDP, Integrated Islet Distribution Program; N/A, not available; OPO, *Organ Procurement Organization*

Table S2. Markers used for cell type annotation

Cell type	Gene marker(s)
α cell	*GCG*
β cell	*INS*
δ cell	*SST*
γ cell	*PPY*
ε cell	*GHRL*
Acinar	*PRSS1*
Ductal	*KRT19*
Stellate	*PDGFRB, COL1A1*
Endothelial	*PECAM1*
Immune	*HLA-DRA*

Table S3. Antibodies used for immunohistochemistry and flow cytometry

Antigen/Conjugate	Species	Source	Catalog #	Application	Dilution
ARX	Sheep	R&D Systems	AF7068	IHC (1º)	1:2000
C-peptide	Rat	Developmental Studies Hybridoma Bank	GN-ID4	IHC (1º)	1:200
NTPDase3 (CD39L3)	Mouse	J. Sévigny	N/A	FC (1º)	1:50
Glucagon	Mouse	Abcam	ab10988	IHC (1º)	1:100
HPa3 (HIC3-2D12)	Mouse	P. Streeter/M. Grompe	N/A	FC (1º)	1:200
HPi1 (HIC0-4F9) – biotin	Mouse	Novus	NBP1-18872B	FC (1º)	1:100
MAFA	Rabbit	Novus	NBP1-00121	IHC (1º)	1:250
MAFB	Mouse	R&D Systems	MAB3810	IHC (1º)	1:1000
Mouse Ig – APC	Goat	BD Biosciences	550826	FC (2º)	1:500
Mouse IgG – Cy3	Donkey	Jackson Immunoresearch	715-165-150	IHC (2º)	1:500
Mouse IgG – Cy5	Donkey	Jackson Immunoresearch	715-175-150	IHC (2º)	1:300
Mouse IgM – PE	Goat	Jackson Immunoresearch	115-116-075	FC (2º)	1:1000
Rabbit IgG – Cy3	Donkey	Jackson Immunoresearch	711-165-152	IHC (2º)	1:500
Rabbit IgG – Cy5	Donkey	Jackson Immunoresearch	711-175-152	IHC (2º)	1:300
Rat IgG – Cy2	Donkey	Jackson Immunoresearch	712-225-153	IHC (2º)	1:500
Sheep IgG – Cy2	Donkey	Jackson Immunoresearch	713-225-147	IHC (2º)	1:500
Streptavadin – BV420	N/A	BD Biosciences	563259	FC (2º)	1:500

1º, primary antibody; 2º, secondary antibody; FC, flow cytometry; IHC, immunohistochemistry

Table S4. Human Pancreatic Tissue Donor Information

Checklist for Reporting Human Islet Preparations Used in Research
Adapted from Hart NJ, Powers AC (2018) Progress, challenges, and suggestions for using human islets to understand islet biology and human diabetes. Diabetologia https://doi.org/10.1007/s00125-018-4772-2.

**Pancreatic tissue**	**6**	**7**	**8**
Unique identifier	DON54	DON42	DON61
Donor age (years)	20	35	55
Donor sex	Male	Male	Male
Donor BMI (kg/m^2)	19.442	26.852	35.565
Donor HbA1c (ng/mL)	5.6	5.1	Not recorded
Origin/source of tissue	IIAM	IIAM	IIAM
Donor history of diabetes? Yes/No	No	No	No
Donor cause of death	Head trauma	Head trauma	Cerebrovascular/Stroke
Glucose-stimulated insulin secretion or other functional measurement	Not done	Not done	Not done
Warm ischemia time (hours)	DBD	DBD	No
Cold ischemia time (hours)	6.2	15.1	26.6

ADI, Alberta Diabetes Institute; DBD, Donation after Brain Death (minimal warm ischemia time); IIAM, International Institute for the Advancement of Medicine
